# Review: advancements in fibrous aerosol filtration across experimental analysis, numerical modeling, and fabrication technologies

**DOI:** 10.1007/s10853-026-13269-8

**Published:** 2026-07-27

**Authors:** Mirza Muhammad Zaid, Ashish Ranjan Kumar, Ashish Kakoria, Lina Zheng, Guang Xu

**Affiliations:** 1 Department of Mining Engineering, Missouri University of Science and Technology, Rolla, MO 65401 USA; 2 Department of Energy and Mineral Engineering, Pennsylvania State University, University Park, USA; 3 School of Safety Engineering, China University of Mining and Technology, Xuzhou, People’s Republic of China; 4 Institute of Occupational Health, China University of Mining and Technology, Xuzhou, People’s Republic of China

## Abstract

The increasing incidence of aerosol-related pulmonary diseases has intensified the need for efficient fibrous filtration technologies capable of capturing ultrafine particles with low pressure drop. Although nonwoven fibrous filters are widely used in medical, industrial, and ventilation systems, their complex internal structures and multiscale transport mechanisms remain insufficiently understood. This review examines the theoretical foundations of aerosol filtration, including single-fiber capture mechanisms and the concept of the most penetrating particle size. In addition, electrostatic interactions, including charge generation, transport, and stability, are incorporated to extend conventional mechanical filtration frameworks. Particular attention is given to flow–particle interactions within fibrous media. We then discuss current approaches for constructing three-dimensional representations of filter structures and highlight how structural assumptions influence the reliability of numerical simulations. Computational fluid dynamics modeling, particle tracking, deposition modeling, and clogging simulations are critically evaluated, with emphasis on commonly adopted assumptions such as homogeneity and steady-state flow. Experimental methodologies for filtration testing are also reviewed, including aerosol generation, particle measurement, and pressure-drop characterization. Instrumentation limitations and measurement uncertainties are discussed. Finally, recent advances in fiber fabrication techniques, including electrospinning, meltblowing, bubble electrospinning, and supersonic solution blowing, are summarized. These fabrication approaches are further analyzed through a material–process–performance perspective, linking fiber morphology and charge behavior to filtration efficiency. The interplay between structural parameters, operating conditions, and aerosol characteristics is analyzed to clarify their combined influence on filtration performance. By identifying unresolved modeling limitations and discrepancies between theoretical predictions and real-world conditions, this review highlights key gaps in current understanding. It integrates filtration theory, electrostatic effects, and fabrication-induced material properties into a unified framework. This approach enables more predictive design of next-generation fibrous filters.

**Graphical abstract:**

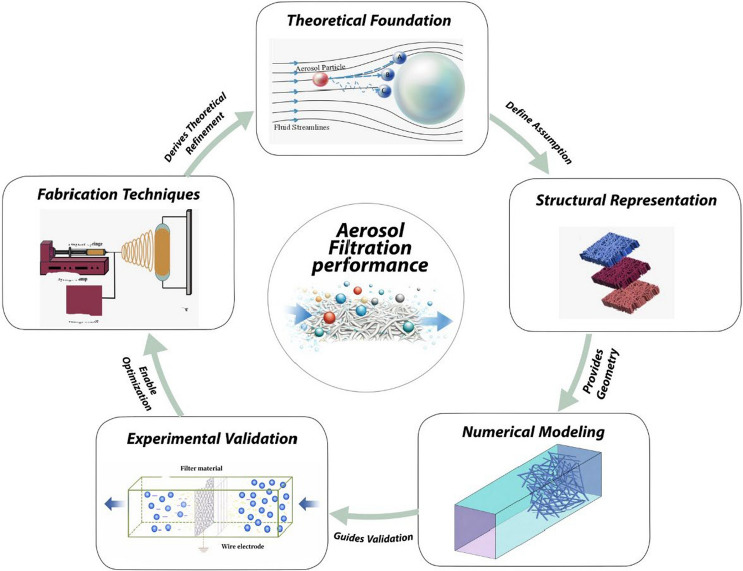

## Introduction

Aerosols are airborne particles that are formed by natural or artificial processes and are released into the environment [1]. Aerosol generation in the environment can occur due to various reasons. Natural sources like volcanic eruptions, wildfires, dust storms, plant emissions, microbial activity, and atmospheric processes like nucleation release aerosols in the atmosphere. Similarly, human activities like industrial processes, transportation, burning fossil fuels, and waste incineration are responsible for aerosol production in the environment. These particles may exist in different forms such as mist, fog, smog, etc. The lighter particles remain suspended in the ambient atmosphere for a long time, could be respirable, and may pose serious threats to human health as well as the climate [2–4]. Therefore, it is necessary to implement effective remedial measures to lower their concentration at the generation source and to capture them immediately if they are airborne.

Particulate control mechanisms can be categorized into six types depending on their process of particle capture. They include (i) solid plate impaction, (ii) liquid impingement, (iii) centrifugal impaction, (iv) virtual impaction, (v) electric and magnetic filters, and (vi) particulate filtration [5–7]. Among all these technologies, filtration is considered very effective because of its high particulate matter (PM) removal efficacy and the economics of the process. Air filtration has extensive applications in respiratory equipment, diesel engine intake systems, heating, ventilating, and air conditioning (HVAC), and nuclear and mining engineering industries [8–10]. Processes of (i) interception, (ii) inertial impaction, (iii) diffusion, (iv) electrostatic attraction, (v) and gravitational settlement are the most common filtration mechanisms observed while using fibrous filter material [11–17]. These mechanisms depend upon different factors like flow velocity, fiber diameter, and particle size. Diffusion is the most prominent capture mechanism for particles smaller than 0.1 um in diameter, interception and inertial impaction are dominant for a particle size > 0.3 um [18]. While these mechanisms are well established, most existing studies treat mechanical and electrostatic capture processes independently. In practice, electrostatic interactions, including charge generation, transport, and dissipation, play a critical role in enhancing particle capture, particularly in nanofibrous and electret-based filters. However, their integration into predictive filtration frameworks remains limited, highlighting a key gap in current understanding.

Fibrous materials are used most for filter media construction [19]. Knitted fibrous filters, plasma textile, and nonwoven fibrous filters are prevalent [19–23]. Nonwoven fiber filter has advantages due to greater permeability, specific area, and controllable pore size distribution. Glass fibers, synthetic fibers, cellulose fibers, wool fibers, metal fibers, ceramic fibers, microfibers, and nanofibers are important classes of non-fibrous filter strands [24]. The filter media should be designed and evaluated for its particle removal efficacy based on several parameters [2]. Fiber-based filter media are generally produced with a fiber diameter of less than 10 um, and the porosity is usually in the 75–90% range. The dust-holding capacity, the pressure drops across the filter, and the filtration efficiency are the most important parameters. The dust-holding capacity can be defined as the number of particles that are trapped on the filter media at the maximum allowable pressure drop. The filtration efficiency is expressed as the percentage change in particle count as they travel across the filter. Particle characteristics including diameter, density, velocity, and surface charges impact the filtration efficiency. The pressure drop is the difference between the filter inlet and outlet [25].

Modern computer simulation techniques have significantly improved the design of filters that have enhanced particulate capture efficacies. The simulations assist in the determination of the most optimal operating conditions which is expensive and challenging to obtain using laboratory or field experiments. For example, a filter made of pleated cylindrical fibers unit, having elevated resistance to temperature and radiation, was studied to compute the velocity, pressure, and efficiency performance. Reduction in the outer diameter of the filter element and increasing its length were shown to enhance its particle capture efficiency [26]. Another study on fibrous filters used GeoDict software to model flow and efficiency with raw data obtained through the X-ray microtomography technique. The thickness, volume fraction of the solid, and the distribution of fiber sizes were calculated using MATLAB. Simulation findings were validated experimentally [27]. Another research used the FiberGeo module of the GeoDict software. Navier–Stokes equations and Darcy’s law were implemented. This research showed that the capacity of the filter media could be enhanced by optimizing the filter volume distribution along the length of the fiber. This study also reinforced that the fiber microstructure impacts the observable filter performance.

CFD models are particularly useful to study airflow and geometry-related parametric studies. In a study, CFD models were developed to research the performance of multi-fiber filters. Computer models showed an increase in filtration efficacy with an increase in face velocity. The dense sparse structure that showed the best efficacy was further optimized using the CFD models to have fewer tiny fibers at the front [28]. CFD modeling technique was used to improve the performance of a hollow fiber filter. The research suggested that the friction coefficient of the media for the staggered configuration of the cells was about 1.5 times higher. This formed the basis for the redesigning of the filtration system [29]. The addition of other techniques, such as DEM, adds to the capabilities of CFD models substantially. For example, the evolution of flow, pressure drops with the geometry, formation of dendrites, etc., can be studied [30].

After simulation and design, the next challenge is creating filter material. Various methods exist for the fabrication of polymer micro- and nanofibers, including meltblowing, electrospinning, bubble electrospinning, solution blowing, self-assembly, phase separation, drawing, template synthesis, wet spinning, dry spinning, and melt spinning. Among these methods, electrospinning stands out as the most popular due to its relative simplicity and scalability, allowing for the reproducible production of fibers ranging from 1 µm to 100 nm. Solution blowing and supersonic blowing, a process akin to meltblowing, has emerged as the most industrially explored technique for large-scale nanofiber fabrication without significant alteration of textile practices. This method exhibits a production rate comparable to meltblowing, making it a promising avenue for industrial applications. Despite these advancements, fabrication techniques are often discussed independently from filtration performance. The relationship between fabrication process, resulting fiber morphology, charge characteristics, and filtration efficiency remains insufficiently explored. Establishing such material–process–performance relationships is essential for developing predictive filter design strategies.

Despite perceptions of industry maturity, the dynamic nature of research and the evolving challenges in air quality maintenance continue to stimulate scientific inquiry and innovation in air filtration technologies. For example, advancements in materials science and technology have led to the development of innovative filtration materials and techniques, such as triboelectric air filters [31], composite nanofiber filters [32], and biobased nanofibrous membranes [33]. These materials offer enhanced filtration efficiency, reduced pressure drop, and improved durability, which have spurred research interest in their applications in air filtration systems. Furthermore, the growing emphasis on sustainability and environmental impact has prompted research into eco-friendly filtration solutions, including biodegradable filter fabrics [34] and green electrospinning techniques [35]. These initiatives align with the global shift toward sustainable practices and green technologies, encouraging researchers to explore environmentally friendly alternatives in the filtration industry. Additionally, public health challenges, such as the COVID-19 pandemic, have underscored the importance of air quality and filtration efficacy. Studies evaluating the effectiveness of air filtration units in reducing exposure to respiratory aerosols [36], as well as the development of high-efficiency particulate matter removal membranes [37], highlight the urgency in addressing air quality concerns and enhancing filtration performance across various settings. Moreover, the complexity of air filtration systems and the diverse range of pollutants they encounter necessitate ongoing research to optimize filtration properties, including pore structure design [38], material selection [39], and performance control strategies [40]. These studies aim to improve filtration efficiency, durability, and adaptability to different environmental conditions, underscoring the need for continuous research and innovation in air filtration.

**Figure 1:**
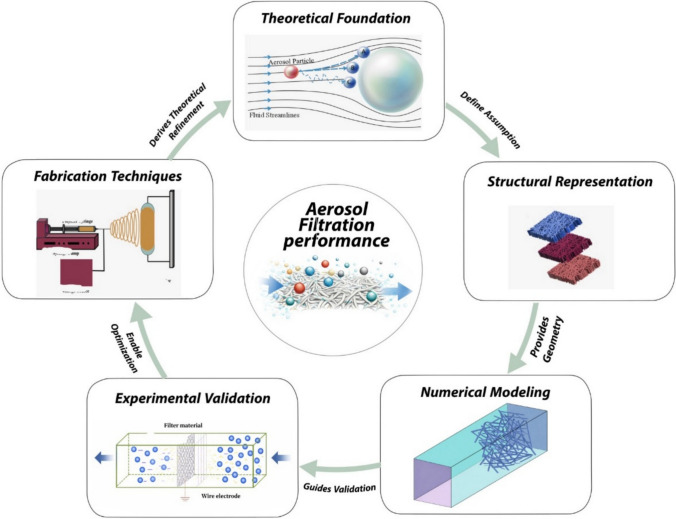
Integrated framework linking theory, structure, fabrication, modeling, and experimentation in aerosol filtration research. Each component contributes to filtration performance metrics including efficiency, pressure drop, quality factor, and most penetrating particle size.

This review paper provides a comprehensive overview of aerosol filtration mechanisms, including theoretical principles, numerical simulations, fiber production techniques, and experimental methods, as illustrated in Fig. 1. Beyond summarizing existing knowledge, the review integrates these aspects into a unified framework to improve understanding of filtration behavior. Particular emphasis is placed on the coupling of mechanical and electrostatic interactions, as well as the influence of fabrication-induced material properties on filtration performance. The role of three-dimensional structural representation and advanced numerical modeling is critically evaluated to highlight limitations in current simulation approaches. In addition, experimental methodologies are examined to identify sources of uncertainty and gaps between idealized models and real-world performance. By linking theory, simulation, and material design, this review identifies key challenges and outlines future directions toward predictive and high-performance fibrous filtration systems.

## Aerosol filtration theories

Establishing a flow field around the fiber is critical to the analysis of the filtration mechanism. The potential flow theory could be used to isolate the flow field around a single fiber perpendicular to the flow direction [41–43]. The velocity distribution along the x-axis for the potential flow is given in Eqs. 1, 2, where *U*_*x*_ is the velocity component along the x-axis and *R*_*f*_ is the fiber diameter. Similar equations could be developed for flow along other axes.

1 $$U_{x} = U\left[ {1 + \frac{{r_{f}^{2} \left( {y^{2} - x^{2} } \right)}}{{\left( {x^{2} + y^{2} } \right)^{2} }}} \right]$$

2 $$U_{y} = - U\left[ {\frac{{2r_{f}^{2} xy}}{{\left( {x^{2} + y^{2} } \right)^{2} }}} \right]$$

**Figure 2:**
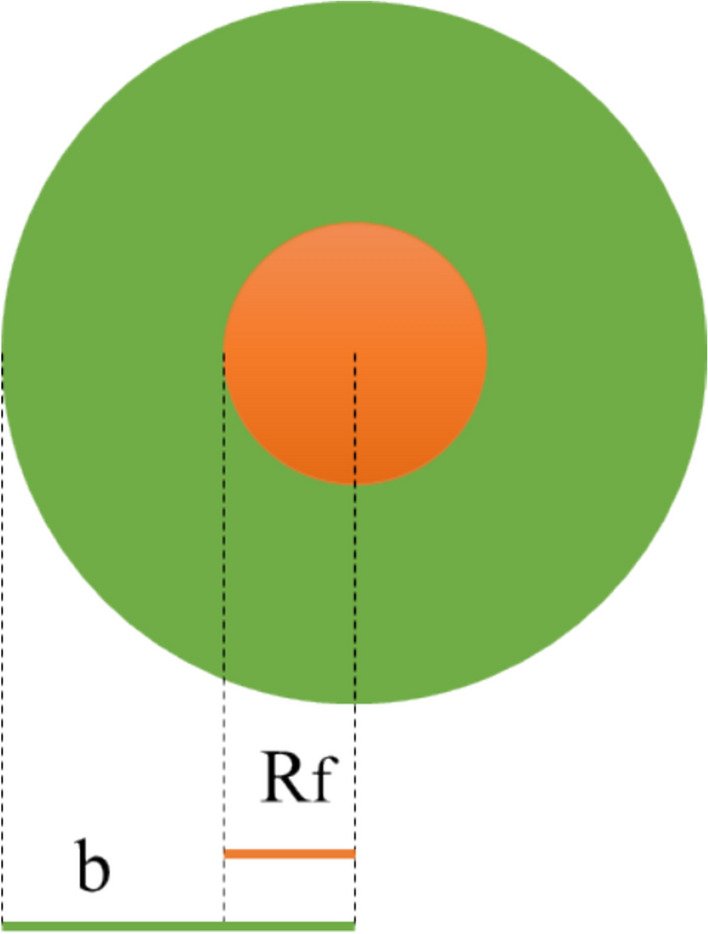
Illustration of a cell model.

The flow field around the fiber is impacted by the viscosity at a very low Reynolds number. So, viscous models are incorporated when applied to the Navier–Stokes equation [44]. However, these models exhibit better accuracy when Reynold’s number is lower than 1.0. The impact of the neighboring fiber on the flow field distribution was considered in the Kuwabara cell model. The cell model can be explained by assuming that a spherical fiber is placed inside a finite sphere. The fiber with a radius *R*_*f*_ and the sphere enveloping the fiber has a radius *b* as shown in Fig. 2.

In the cell model, the flow field around all the fibers is the same and these fibers are placed perpendicularly to the direction of the main flow. This simplified assumption and the geometry of the cell model were adopted by many researchers in the literature [35, 36]. The utilization of the cell model’s geometric properties enables the calculation of the flow pattern around fiber, which is critical to further transportation models [45, 46]. In the Kuwabara cell model, the packing of the fiber is in a hexagonal pattern. Unlike Happel flow cell model, Kuwabara cell model considers the vorticity at the boundary to be zero. The stream function allows us to visualize and understand the formation of vortices and the behavior of the fluid flow in the wake of an obstacle or disturbance. The stream function, ψ, for the cell model is given by Eq. 3:

3 $$\Psi = \frac{vr}{{2Ku}}\left\{ {2\ln \frac{r}{{R_{f} }} - 1 + \alpha + \frac{{R_{f}^{2} }}{{r^{2} }}\left( {1 - \frac{\alpha }{2}} \right) - \frac{\alpha }{2}\frac{{r^{2} }}{{R^{2} }}} \right\}\sin \theta$$

where v = $\frac{V}{1-\alpha }$, which is the average velocity in the filter and ku = −0.5lnα−0.75 + *α*−0.25${\alpha }^{2}$, also called the Kuwabara hydrodynamic factor.

The particle collection performance of the fibrous filters can be determined using the single-fiber efficiency (SFE). SFE can be defined as the percentage of the particles trapped on the fiber to the total number of particles introduced [18]. As a single fiber is considered the smallest unit of a filter media, the collective result of a single fiber is used to calculate the overall efficiency of the filter media. Equation 4 below could be used to calculate the collection efficiency of a filter media from a single‐fiber efficiency *η* [7, 47].

4 $$E = 1 - \exp \left( {\frac{ - 4\alpha \eta t}{{\pi d_{f} \left( {1 - \alpha } \right)}}} \right)$$

**Figure 3:**
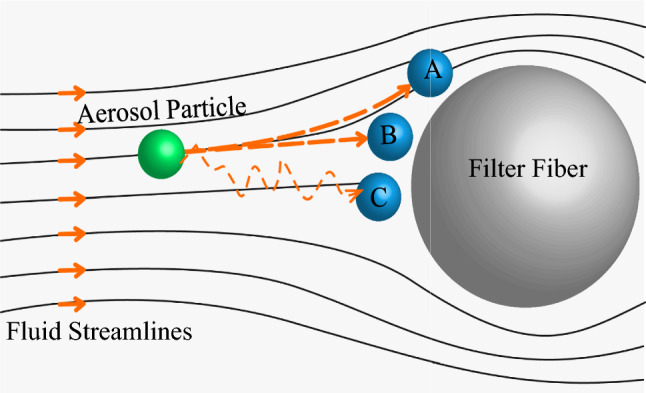
The illustration of basic filtration phenomena **A** interception, **B** inertial impaction, and **C** Brownian diffusion.

The major filtration mechanisms for the removal of aerosol particles are interception, diffusion, and inertial impaction as reported earlier [48, 49]. As shown in Fig. 3 (see the trajectory of particle A), the interception mechanism assumes that the particles are captured by the fiber when the distance between the surface of the fiber and the center of the particle is less than the fiber radius [42, 43, 50]. The interception can remove the particles larger than 0.6 um [51]. The single-fiber efficiency due to interception is given by Eq. 5:

5 $$\eta_{R} = \frac{y}{{R_{f} }}$$

where ${R}_{f}$ is the fiber diameter and y is the distance between two streamlines. For any point in a cell model, the stream function ψ = *vy,* so the SFE due to interception is given by Eq 6.

6 $$\eta_{R} = \frac{y}{{R_{f} }}$$

Let θ s= $\frac{\pi }{2}$ and r = *R*_*f*_ + *R*_*p*_ in the stream function, then filtration efficiency for Kuwabara flow field is given by Eq 7:

7 $$\eta_{R} = \frac{1 + R}{{2Ku}}\left[ {2\ln \, \left( {1 + R} \right) \, - 1 + \alpha + \left( {\frac{1}{1 + R}} \right)^{2} \left( {1 - \frac{\alpha }{2}} \right) - \frac{\alpha }{2}\left( {1 + R} \right)^{2} } \right]$$

where *α* is fiber volume fraction, and it has a wide range for the fiber material [18]. Lee and Liu proposed the approximation for the SFE due to interception, which has been used by many researchers and is given in Eq. 8.

8 $$\eta_{R} = \frac{1 - \alpha }{K}\frac{{R^{2} }}{1 + R}$$

In cases where particle size exceeds 1.0 μm, particle behavior is notably influenced by their inertia. In such instances, these larger particles deviate from following the streamline direction within the fluid flow and captured by fiber surface due to inertial impaction (see the trajectory of particle B in Fig. 3) [7]. Another useful dimensionless parameter employed in characterizing the efficacy of inertial impaction is the Stokes number, denoted as Stk. Typically, it is defined as the ratio between the particle relaxation time and the fluid characteristic time given in Eq. 9. Equation 10 is used for smaller stokes number, where *J *= 29.6−28α^0.62^. Equation 11 is used for larger stokes number where *K*_1_ is defined as a constant which is dependent on the flow field. For the Kuwabara flow field, its value is 0.085 with α = 0.05.

9 $$Stk = \frac{tV}{{d_{c} }}$$

10 $$\eta I = \frac{J.St}{{\left( {2Ku} \right)^{2} }}{\text{for small }}St$$

11 $$\eta I = 1 - \frac{{k_{1} }}{St}{\text{for large }}St$$

where *t *is the relaxation time of the particle, which is defined as the time taken by the particle to adjust its velocity to new conditions of force and is given by Eq. 12. The parameter *C*_*c*_ is the Cunningham slip factor which is given by, while *Kn*_*p*_ is the Knudsen number.

12 $$t = \frac{{\rho_{p} d_{p}^{2} C_{c} }}{18\mu }$$

13 $$C_{c} = \, 1 + kn_{p} \left( {1.257 + 0.4e^{ - 1.1/kn}_{p} } \right)$$

When the particles’ sizes are very small, they deviate from defined streamlines. Their random motion causes them to be captured by fiber via diffusion, resulting in increased single-fiber efficiency [18]. The Brownian diffusion is shown in Eq. 13 (see trajectory of particle C). The single-fiber efficiency by diffusion is given in Eq. 14 where *K* is the hydrodynamic factor of Kuwabara flow and is given by Eq. 15 where Pe is the Peclet number.

14 $$\eta_{D} = 2.6\left( {\frac{1 - \alpha }{K}} \right)^{1/3} Pe^{ - 2/3}$$

15 $$K = - \frac{1}{2}\ln \alpha - \frac{3}{4} + \alpha - \frac{1}{4}\alpha^{2}$$

The overall single-fiber efficiency due to interception, η_*R*,_ due to this mechanism is given by Eq. 16, where η_*D*_ is SFE due to diffusion and η_*I*_ is the SFE due to inertial impaction.

16 $$E = 1 - \left( {1 - \eta R} \right)\left( {1 - \eta D} \right)\left( {1 - \eta I} \right)\left( {1 - \eta DR} \right)$$

The single-fiber efficiency theory used to describe the filtration process assumes homogeneity in the filter media. However, the fibrous filter material structure is complicated with random arrangement of fibers of different diameters. The airflow direction may not be perpendicular to the fiber. Therefore, these models do not accurately predict the pressure drop and flow field in realistic filter geometries. While mechanical capture mechanisms govern particle removal in fibrous media, their effectiveness diminishes near the most penetrating particle size. In such regimes, electrostatic interactions provide an additional pathway for enhancing filtration performance.

### Electrostatic interactions in fibrous aerosol filtration: mechanisms, fabrication, and stability

Electrostatic interactions play a critical role in fibrous aerosol filtration, particularly for submicron particles where conventional mechanical capture mechanisms become less effective. While diffusion, interception, and inertial impaction govern particle capture in fibrous media, their efficiency decreases near the most penetrating particle size (MPPS), necessitating additional mechanisms to enhance filtration performance. In practical applications, electrostatic effects have been widely utilized in electret-based fibrous filters, which are extensively employed in heating, ventilation, and air conditioning (HVAC) systems, industrial air purification, and personal protective equipment such as respirators and surgical masks [52]. These systems achieve high filtration efficiency without significantly increasing pressure drop, thereby overcoming the fundamental trade-off between airflow resistance and particle capture.

The electrostatic capture of particles is primarily governed by Coulombic and dielectrophoretic forces. Coulombic attraction occurs between charged particles and fibers and is proportional to the electric field strength, while dielectrophoretic forces act on neutral but polarizable particles in non-uniform electric fields and depend on the gradient of the squared electric field. These mechanisms enable efficient capture of both charged and neutral particles and are particularly important in the submicron regime [52, 53]. The effectiveness of electrostatic filtration is therefore controlled not only by the magnitude of charge but also by the spatial distribution of the electric field around fibers.

In polymer electret filters, electrostatic effects originate from quasi-permanent charges stored within the fiber matrix. These charges exist as surface charge, space charge, and dipolar polarization and are typically trapped in molecular chains, crystalline–amorphous interfaces, or heterointerfaces within the material. However, macroscopic surface potential measurements do not fully represent microscopic charge distribution, highlighting a key limitation in understanding electrostatic behavior. Advanced nanoscale characterization techniques such as Kelvin probe force microscopy have revealed strong local variations in surface potential and charge heterogeneity along individual fibers, emphasizing the importance of micro- and nanoscale charge distribution [54, 55].

Electrostatic performance is strongly dependent on fabrication methods and material chemistry [56]. Charges can be introduced through corona charging, triboelectrification, polarization, or electrospinning, each producing distinct charge distributions and stability characteristics [57]. Electrospinning is particularly effective due to in situ charge injection and trapping during fiber formation [58]. In addition, material selection governs charge polarity and distribution. For example, co-electrospinning polymers with different electron affinities generates bipolar charge distributions and interfacial charge hotspots, which enhance internal electric fields and particle capture efficiency [56]. Similarly, engineered bipolar heterocharge systems produce strong electric field gradients that significantly improve electrostatic attraction compared to uniformly charged fibers.

Beyond electret-based systems, intrinsic electrostatic effects can arise from material polarization. Ferroelectric polymers such as PVDF exhibit permanent dipole moments that generate internal electric fields without external charging. The electrostatic behavior of these materials is strongly linked to crystalline phase composition, particularly the *β*-phase, which enhances dipole alignment and particle capture efficiency [59]. External electrical activation can further amplify electrostatic performance. For instance, polarization combined with triboelectric charging significantly increases surface potential and filtration efficiency in PVDF-TrFE nanofibers, demonstrating that electrostatic behavior can be tuned through controlled activation processes [53].

Recent developments have shown that electrostatic effects can also be dynamically generated during filtration. Self-powered filtration systems based on triboelectric and piezoelectric mechanisms convert mechanical energy into electrical energy, enabling continuous charge generation and sustained electrostatic fields during operation [60]. In these systems, airflow-induced deformation and fiber–fiber contact produce charges that maintain filtration efficiency even under conditions where traditional electret filters would experience charge decay. Furthermore, triboelectric nanogenerator‐based systems can generate strong electric fields that ionize surrounding air, producing negative air ions that attach to particles and enhance their deposition. This ion-mediated electrostatic mechanism provides an additional pathway for particle capture beyond fiber surface interactions [61].

The coupling between electrostatic effects and fiber structure plays a key role in determining filtration performance. Multiscale fibrous architectures, including bimodal fiber distributions and three-dimensional porous networks, enhance both mechanical and electrostatic capture [62]. Electrostatic forces promote more uniform particle deposition across the filter depth, reducing localized clogging and improving dust-holding capacity and service life [63]. However, excessive fiber packing can lead to electrostatic interference between fibers, reducing field strength and diminishing capture efficiency [64]. Therefore, optimization of both structure and charge distribution is essential for maximizing filtration performance.

A major challenge in electrostatic filtration is maintaining charge stability under realistic operating conditions. Environmental factors such as humidity, temperature, and particle composition can accelerate charge dissipation and reduce electrostatic capture efficiency [62, 63, 65, 66]. Hygroscopic particles and high relative humidity conditions can significantly degrade performance due to combined effects of charge decay and altered deposition behavior [63]. Recent approaches address this limitation through the use of deep charge traps, which improve charge stability, and surface engineering strategies that reduce moisture adsorption and maintain electrostatic performance [54, 62, 65].

Despite significant advances, a comprehensive understanding of electrostatic field evolution, charge transport, and their coupling with particle dynamics remains incomplete. In particular, the relationship between fabrication-induced charge states, nanoscale charge distribution, and macroscopic filtration performance is not fully resolved. Current models often assume simplified charge distributions and do not account for dynamic interactions between airflow, electrostatic fields, and particle deposition. Addressing these challenges requires integrated experimental and computational approaches that combine multiscale charge characterization with coupled multiphysics modeling. Such efforts are essential for the predictive design of next-generation fibrous filters with improved efficiency, stability, and energy performance.

### Emerging theoretical directions beyond classical fibrous filtration theory

While classical single-fiber theory describes aerosol capture through interception, diffusion, and inertial impaction in an idealized hydrodynamic field, recent studies suggest that this framework is insufficient for charged fibrous media. In electroactive filters, particle motion is governed by coupled interactions among gas flow, electric field, and particle transport, and electrostatic contribution depends not only on charge magnitude but also on electric field gradients, charge configuration, and charge stability. Recent work on bipolar heterocharged nanofibers shows that non-uniform-charge distributions generate stronger field-intensity gradients and stronger electrostatic attraction than simplified uniform-charge assumptions. In parallel, studies on electrospun and electroactive nanofibers indicate that deep-trapped charges, interfacial polarization, and in situ charge generation during fabrication strongly affect long-term filtration performance, particularly under humid conditions. These developments point toward an emerging multiphysics view of aerosol filtration in which hydrodynamics, electrostatics, charge transport, and nanoscale surface potential distribution must be considered together rather than as separate effects.

#### Electrostatic–hydrodynamic coupled transport mechanism

Classical single-fiber theory treats particle capture as a result of interception, diffusion, and inertial impaction in a prescribed flow field [67]. While this framework provides a useful baseline, it assumes that particle transport is governed solely by fluid mechanics and neglects additional forces present in charged systems. In electret filters, however, particle motion is influenced by the combined effects of gas drag, Brownian motion, and electrostatic interactions, resulting in more complex transport behavior. Recent studies have emphasized that realistic electrostatic filtration cannot be accurately described without simultaneously accounting for the coupled interactions between fluid flow, electric fields, and particle dynamics, particularly because the electrostatic field evolves over time due to charge migration, surface dissipation, and particle deposition. These insights indicate that the next generation of filtration theory must move beyond isolated single-fiber efficiency formulations toward a three-field coupled transport framework that integrates flow, charge, and particle transport into a unified model [52, 68, 69].

#### Field-gradient‐controlled capture mechanism

A key advancement in recent electret filtration research is the recognition that particle capture is governed not only by the magnitude of charge on fibers but also by the spatial distribution and gradients of the surrounding electric field. Earlier studies have described electrostatic capture through Coulombic and dielectrophoretic mechanisms, demonstrating that the dielectrophoretic force acting on neutral particles is directly dependent on the gradient of the squared electric field [52]. More recent work has extended this understanding by showing that bipolar heterocharge configurations generate stronger electric field gradients compared to uniformly charged systems, leading to enhanced electrostatic attraction and improved filtration efficiency. These findings support a revised theoretical framework in which particle capture is dictated by electric field topology and gradient intensity, rather than by average surface potential alone [54, 70].

#### Charge-configuration mechanism for fibrous filters

Traditional filtration models often assume uniform or simplified charge distributions along fibers; however, recent studies indicate that this assumption is overly simplistic for electroactive fibrous systems. A recent study demonstrated that charge configuration itself can be treated as a critical design parameter rather than a fixed material property. Their PS/PVDF-HFP system shows that bipolar heterocharge distributions significantly outperform unipolar configurations, as oppositely charged neighboring fibers generate stronger electric field gradients and enhance electrostatic attraction toward airborne particles. These findings suggest that filtration performance is governed not only by conventional parameters such as fiber diameter and packing density but also by the spatial distribution of charge within and between fibers, highlighting the importance of charge architecture in advanced filtration designs [54, 71]

#### Deep-trap and charge-retention mechanism

Another important development in electrostatic filtration is the shift from evaluating performance based solely on initial charge density to considering the role of trap depth and charge stability. In electret materials, charges are stored in energy traps within the polymer matrix, and the depth of these traps governs how easily charges can be released under environmental or operational conditions [72, 73]. One study highlighted that electrospinning can promote the formation of deeper charge traps compared to conventional charging methods, leading to improved charge retention. Similarly, another research demonstrated that deep-trapped charges remain stable even under high humidity conditions, directly contributing to sustained filtration efficiency [74, 75]. Charges located in shallow traps are more susceptible to dissipation through moisture, thermal activation, or particle interaction, whereas deep-trapped charges require higher activation energy for release and therefore exhibit greater stability. As a result, long-term filtration performance becomes a function of trap depth distribution, charge mobility, and environmental exposure. This leads to a transition from conventional “initial charge-based” models toward trap-governed electrostatic durability models, where understanding charge transport pathways and retention mechanisms is essential for designing stable and high-performance fibrous filters [63, 73, 76].

#### Fabrication-induced charge dynamics and interfacial electroactive filtration

Electrospinning-based systems introduce an important theoretical advancement by treating fiber formation as a dynamic process of charge generation and decay rather than a purely geometric fabrication method. During electrospinning, multiple charge sources exist simultaneously, including ion migration within the solution, localized corona-like effects, and interactions with ionized surrounding air, while competing charge dissipation mechanisms occur during jet stretching and after fiber deposition. As a result, the electroactivity of the resulting fibers is governed by a balance between charge generation, transport, stabilization, and decay during fabrication. In addition, material composition further enhances electrostatic behavior through interfacial polarization and conductivity-assisted mechanisms [75, 77]. For example, the incorporation of Ti₃C₂Tₓ MXene increases electrical conductivity and promotes interfacial polarization, strengthening particle–fiber interactions beyond classical mechanical capture. Enhanced filtration performance in such systems is attributed not only to reduced fiber diameter but also to increased surface potential, dipole–dipole and induced-dipole interactions, and Maxwell–Wagner–Sillars-type polarization effects [78]. These findings support a broader theoretical framework in which filtration is governed by electroactive material properties and interfacial charge dynamics, rather than solely by fiber geometry and conventional capture mechanisms.

Three-dimensional models that represent geometry accurately could be used to reliably predict filter performance and are discussed in the next section.

## 3D virtual fibrous structures generation

**Figure 4:**
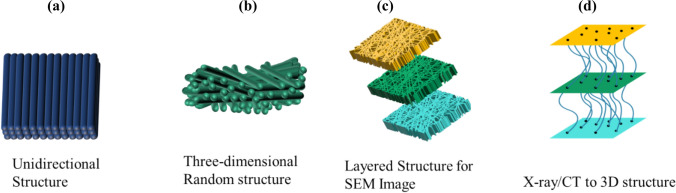
Representative approaches for generating three-dimensional (3D) geometries of fibrous filter media for modeling and simulation. **A** illustrates an idealized unidirectional fiber array composed of parallel cylindrical fibers, while **B** shows a three-dimensional random fiber network with stochastic orientation and spatial distribution. **C** represents a layer-by-layer reconstruction derived from stacked SEM image slices, and **D** depicts a volumetric microstructure reconstructed using X-ray or computed tomography (CT) imaging techniques.

Accurate virtual 3D structure of the filter media is critical to precisely simulate results from the computer models [19]. Many modern technologies like magnetic resonance imaging (MRI), X-ray tomography, and synchrotron imaging are used to capture the structure of the filter media [79]. These imaging technologies, however, are limited by their inability to capture the entire filter domain when a higher resolution is desired for an accurate analysis of the fiber network. X-ray tomography and synchrotron imaging are most suited for microfiber structure and are limited to capturing nanofiber structure [80]. These techniques capture a series of 2D slices or sections and then reconstruct them into a 3D volume image through computer processing [81]. This problem can be addressed by generating 3D virtual geometries of the filter media using 3D modeling software. In recent years, GeoDict, Blender, Voxel, CAD, and several other software have been used to generate the 3D virtual geometry of filter media [82–85]. Different 3D media generation techniques are shown in Fig. 4.

**Figure 5:**
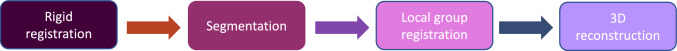
Algorithms are used for determination and connection of centerline pixels for images obtained from SEM, MRI, and X-ray tomography.

One technique to create the 3D filter geometry is to draw straight fibers in Cartesian coordinates by placing the fibers randomly in the flow domain shown in Figs. 4a, 5b. Generally, the fibers are considered perfect cylinders with specific diameters. This helps in generating the homogeneous virtual geometry of the filter media. The parameters needed for this method are fiber diameter (*D*_*f*_), porosity (*ɛ*), and the analysis volume size [50, 86]. A single fiber is then placed in the Cartesian plane with random orientations. The procedure is repeated by varying parameters including fiber diameter and porosity until the required packing density or solid volume fraction is obtained [84]. This method has been successfully used to calculate the pore size distribution (PSD) from the inscribed sphere generated by these fibers [87]. By decreasing the porosity, the PSD curve becomes broader. This leads to decreased voids due to increased fiber count in the flow domain. With an increase in fiber diameter for a fixed porosity value, the PSD curve moves to the right. The shape remains unchanged indicating that thinner fibers lead to smaller void pores representing the porosity. This method is advantageous as it can generate different networks of pores to be used in pore-scale modeling of trickle bed reactor, paper coating layers, and others [53, 54].

The second technique for filter geometry construction is to mimic the microstructure of the filter media from the images obtained from scanning electron microscope (SEM) as shown in Fig. 4c [88]. The filter material image is first converted into a black and white image. The appropriate grayscale value for the image has been suggested in the previous research [89]. The next step is to determine the centerline pixel of the fiber to determine the connectivity of the fiber map [90]. The algorithm for determination and connection of the centerline of the pixel has five major steps and is shown in Fig. 5. A Gaussian filter is applied first to remove the background noise in the first step through a process called rigid registration. The boundaries of the domains of interest are defined in the next step called segmentation. Identical lines are connected and contoured in the next step called contour grouping. In the local group registration, various contoured groups are joined and are converted into 3D lines. These contours are aligned using both rigid and local group registration techniques, enabling the accurate grouping and subsequent reconstruction of the 3D structures [91]. Generally, only the top layer of the fiber is utilized to regenerate a structure like the actual media [84]. The tracing is continued and now includes the neighboring fibers. This process is repeated for each visible fiber on the surface of the filter media. To generate the 3D shape of each fiber, a circle of a known diameter is generated on the centerline, and this circle is extruded to generate a tube-like shape [92]. This procedure will generate stacked layers of fiber media. The layer spacings and the fiber diameters are adjusted so that the packing density of the virtual 3D model is equal to the real filter media.

The third filter geometry construction technique is constructing 3D images of fiber from an X-ray image. After the X-ray image is obtained, the cross-sectional layers are cut. Each layer shows the fiber dots which pass through it. Different layers are stacked above each other. Then these dots on these layers are connected and generate a realistic 3D geometry of the filter media. Stochastic geometry modeling is the fourth method that could be used to generate realistic models of filter media including the microstructures of the material [93]. Once parameters like porosity, fiber diameter, etc., are chosen, the layers are created and stacked over each other for the 3D generation of models of the media for CFD simulations [93]. Top and bottom images are used to determine the properties like fiber diameter while the orientation of fibers, pore size distribution, and the thickness of the medium are better understood by X-ray computer tomography [94]. However, SEM, X-ray tomography, and MRI techniques sometimes could lead to erroneous results due to their dependence on inputs from the images. Other processes like image tracing, changing the resolution, and converting the image into grayscale might have an effect in mimicking the actual internal structure of the filter media.

A fifth method by using some contemporary programming languages including Python and Fortran along with blending techniques could be used to generate the 3D geometry of the fibers. Three-dimensional geometry of the filter media is generated first. Fiber diameter, packing density, length of the filter element, the simulation domain, etc., are specified. The single fiber is rotated along the x-axis, and stresses are applied to see the material yield. In the second step, the single-fiber model is turned into a faceted fiber using the same method, other fibers are generated to create a complete 3D geometry of the filter media until the required packing density is reached [12, 15, 19, 61]. The major advantage of this method lies in its ability to generate any geometrical shape.

## Numerical simulation methods

### Flow field simulation

**Figure 6:**
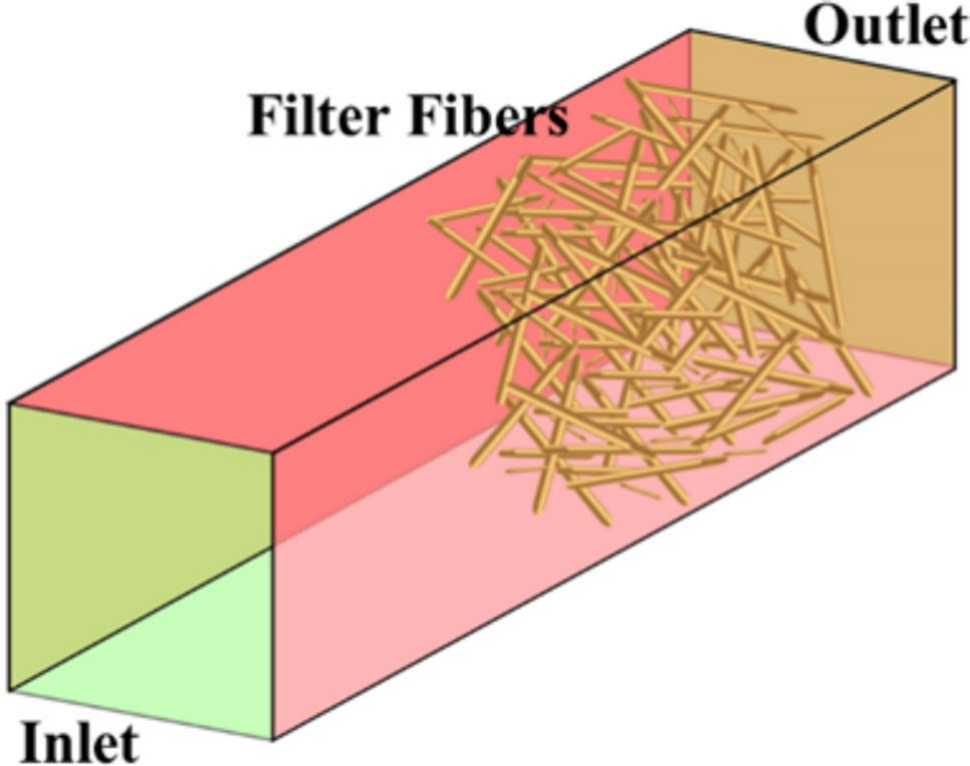
Simulation domain and boundary conditions for virtual filter media.

CFD modeling is a powerful simulation tool that could be used to develop the flow field around the fibers positioned in a flow domain. Velocity vectors and contours, pressure, and other contours, and streamlines of flow in addition to several other fluid flow parameters could be derived from the CFD models. The continuity equation that describes the conservation of mass through the flow domain (Eq. 17) and momentum equations (Eq. 18) are solved numerically to determine the airflow field inside the airflow domain as shown in Fig. 6 [95]. The air enters the simulation domain at the inlet, then moves through the simulation domain, and passes through the filter fibers. Air then exits the system from the outlet. For the development of CFD models, the airflow around the fiber strands of the filter is also assumed to be laminar and at a steady state [13, 83, 96]. These conditions allow the generation of high-resolution converged flow fields.

17 $$\frac{\partial u}{{\partial x}} + \frac{\partial v}{{\partial y}} + \frac{\partial w}{{\partial z}} = 0$$

18 $$\frac{\partial p}{{\partial x}} = \mu \left( {\frac{{\partial^{2} u}}{{\partial x^{2} }} + \frac{{\partial^{2} u}}{{\partial y^{2} }} + \frac{{\partial^{2} u}}{{\partial z^{2} }}} \right)$$

The filter geometrical dimensions are critical to the development of models that accurately represent the flow around the fibers and their collection efficacy. Moreover, when the fiber diameters are significantly larger than the mean free path of the air molecules around them, slip is less likely to occur [84]. This further reinforces the assumptions of the filtration theory that the flow around the fibers is continuous. Additionally, as the fiber size diminishes and is comparable to the mean free path of the air molecules, no slip condition does not hold true anymore. Aerodynamic slip becomes dominant at these length scales and is characterized by the Knudsen number *k*_*n*_ = 2λ/*d*_*f*,_ where λ is the mean free path of molecules. A continuum flow regime is assumed for a Knudsen number smaller than 0.001, a slip flow regime in the 0.001–0.250 range, and transient regime for the number exceeding 0.25 [96]. Researchers, however, differ on the impact of slip flow on the fiber surface. One research demonstrates its significant impact on the pressure drop and no influence on filter efficiency [19, 63, 64]. Other research showed a smaller drag force that lowers the pressure drop due to slip. This led to higher airflow near the surface resulting in enhanced collection efficiency [97].

The length of the simulation domain is generally determined by the Brinkman criteria, α, which is a function of the resistance of the porous medium [98]. It is reported in a study that the length of the domain, which is 14 times the Brinkman criteria, is accurate enough for the simulations. Smaller volumes require more repetitions to obtain better results [13]. The Brinkman screening length determines whether to use the Brinkman equation for fluid flow in porous media simulations. It uses the particle size relative to pore size; small particles favor the Brinkman equation, while larger particles may require alternative models. The Brinkman equation used for the flow through the porous media is shown in Eq. 19:

19 $$- \nabla p \, = \, - \mu \nabla^{2} u + \, \mu \alpha^{2} u$$

where p is averaged pressure, u is the averaged fluid velocity, µ is the dynamic viscosity, and *α* is the resistance of the porous medium. The value of α sets the length scale (*α*^−1^), called Brinkman screening length, over which fluid flow decays in a given porous medium [99].

### Particle tracking

Particle tracking in a CFD model involves following the trajectory of transportation of individual particles through a simulation. In a CFD model, particle tracking is typically performed by updating the particle positions based on their velocities and the time step of the simulation. The particle’s motion is governed by drag, gravity, and any other external or internal forces. The Euler method (EL) and the Lagrange method (LM) are two commonly used approaches for particle tracking in fluid dynamics simulations [100]. The Euler method tracks particles by considering them as passive tracers that move with the flow. It assumes that the particles do not influence the fluid flow and their positions are determined solely by the fluid velocity field. In this method, the fluid velocity is interpolated from the grid points, and the particle positions are updated based on this interpolated velocity. The Euler method is relatively computationally efficient. The disadvantage associated with the EL method is that it may not accurately capture particle behavior in highly turbulent flows or situations where particles significantly affect the fluid motion [30, 101, 102]. On the other hand, the Lagrange method treats particles as individual entities with their own positions and velocities. Each particle is tracked independently, and its motion is determined by integrating the governing equations. This method accounts for the particle–fluid interaction, including forces such as drag and lift. The Lagrange method provides more accurate representation of particle behavior in complex flows but can be computationally demanding, especially for a large number of particles.

In the Eulerian–Lagrangian method (ELM), the particles are modeled as a continuous fluid phase that is coupled with the continuous fluid phase, but they do not have the same properties as the fluid. Instead, the particles are modeled as a separate phase with its own properties, such as density and size [103]. In the Euler–Lagrange method, the fluid flow is solved using the Eulerian approach, where the fluid velocity field is computed on a fixed grid. This allows for accurate modeling of the fluid flow and its interactions with the particles. The particles, on the other hand, are treated using the Lagrangian approach, considering them as discrete entities with their own positions and velocities. The Euler–Lagrange method incorporates the advantages of both methods. This method is particularly useful in situations where the interactions between particles and the fluid flow are significant and need to be captured with higher fidelity [104].

The cleaning efficiency of fibrous filters for an aerosol particle of a known size strongly depends on the particles’ diameter. The particles with a larger diameter have larger mass and, therefore, large momentum for a known airflow velocity. The transportation medium, air, for these large particles is considered continuous. However, as the aerosol particle sizes approach sub-micrometer sizes, this assumption is no longer valid. A suitable correction factor is required to be built into the computer models to account for this significant lowering of sizes, which could approach the mean free path of air molecules. This is the path an air molecule travels in a straight line before colliding with another molecule. Using these correction factors allows the accurate determination of the drag forces acting on them and hence the particle trajectories. Cunningham’s slip correction factor, Cc, given by Eq. 20, is the most commonly adopted factor used in scientific calculations where λ and d are the mean free path of air molecules and the particle diameter, respectively [105].

20 $$Cc = 1 + \frac{\lambda }{d}\left[ {2.514 + 0.8 e^{{ - 0.55 \frac{d}{\lambda }}} } \right]$$

This factor approaches unity for large-sized particles and increases exponentially for smaller particles. Millikan’s experiments of 1910 demonstrated its approximate linear dependence on the mean free path of the gas [106, 107]. The data from his experiments continue to be evaluated by researchers and have led to some minor modifications in the original equation [108]. More recently, there have been several accurate determinations of the drag forces leading to precise calculation of slip correction factors. For example, a three-dimensional simulation of incompressible flow around sphere using the full solution of the Navier–Stokes’s equation showed that the slip correction factor also depends on the Reynold’s number in addition to the Knudsen number. This will significantly improve the particle tracking results [109, 110].

### Particle deposition and filter clogging

**Figure 7:**
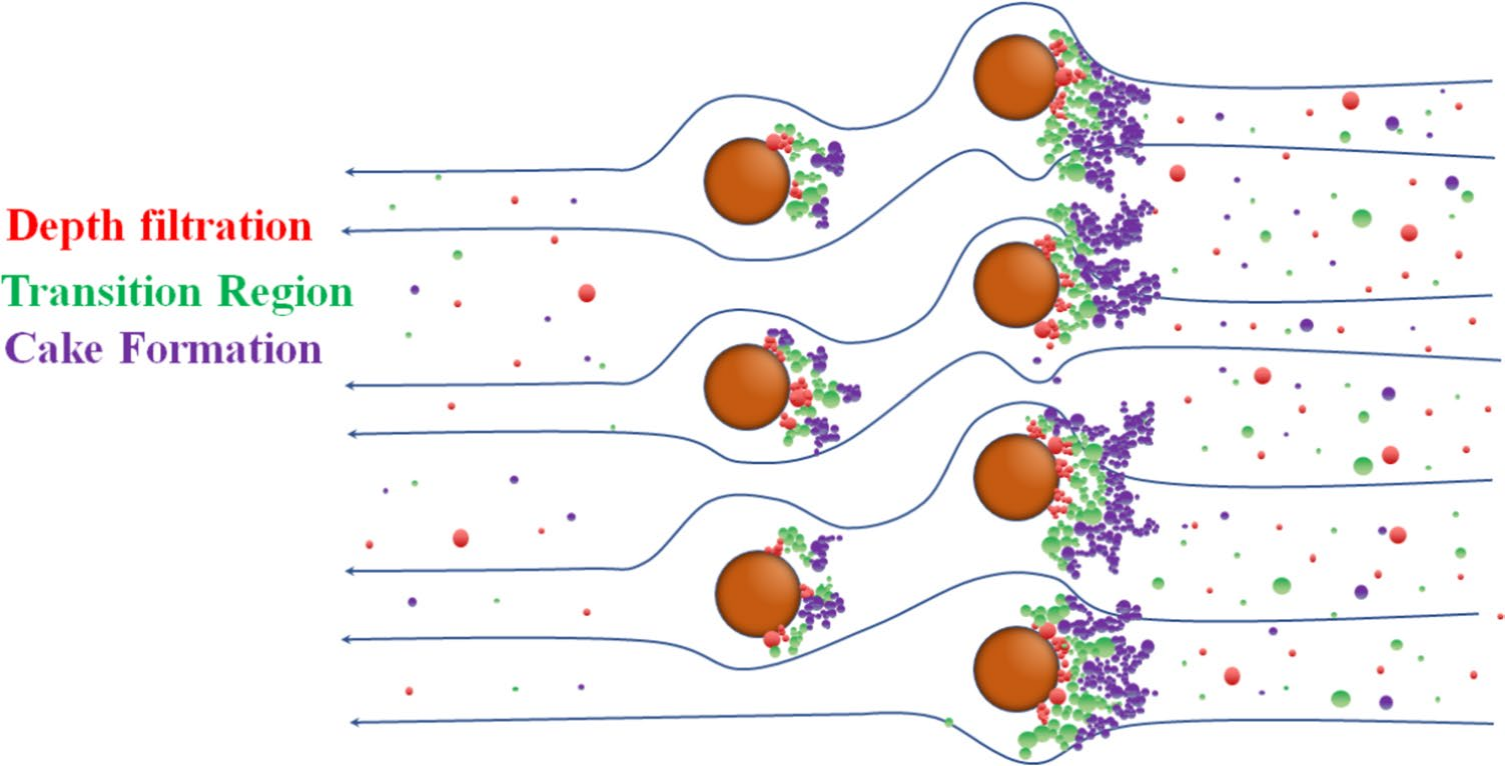
Loading of particles during filtration process, i.e., depth filtration, transition regime, and surface filtration.

The loading of the particles on the filter progresses in three stages: depth filtration, transition regime, and surface filtration as shown in Fig. 7 [2]. The red particles in Fig. 7 demonstrate the depth filtration process. The particles first start to get deposited on the fibers followed by other incoming particles resulting in dendrite formation. This causes an abrupt rise in pressure drop, often up to 80 – 90%, across the filter during the depth filtration [111]. It is important to note that depth filtration captures only a few particles introduced to the filter. A study on nanofiber filters showed that only 3.0% of total mass was captured by depth filtration. Depth filtration, however, lays the foundation for transition and cake filtration. As the particle loading progresses, these dendrites bridge with neighboring dendrites during the transition regime as demonstrated in Fig. 7 by the green particles. These dendrites, then, grow further to form the continuous cake layer across the filter surface capturing all the incoming particles (purple particles in Fig. 7). This stage is called surface filtration or cake formation [112]. Surface filtration and transition regime are more important because of their major contribution to the overall capture. About 25.0% of the aerosol mass is collected in the transition and 72.0% in the surface filtration [112].

The shape of the particle dendrite formed on the fiber depends upon various factors including the mechanism of their deposition and the point of injection. The interception mechanism encourages the particles to get deposited on the lateral side of the fiber. In the case of inertial impaction, the particles usually follow a straight path and get deposited on the front side of the fiber. For Brownian diffusion, the dendrites are formed over the entire surface of the fiber [49]. The random injection of the particles from the airflow inlet results in a realistic dendrite shape formation unlike a single-point injection that leads to unrealistic shapes [113].

**Figure 8:**
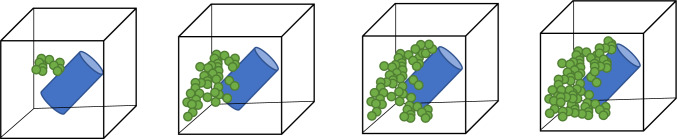
Particle dendrite formation as the simulation progresses.

The modeling of particle capture can be done using computational fluid dynamics (CFD) modeling technique software. The standard discrete phase module (DPM) in the CFD treats the particle as a point mass. Therefore, it is incapable of mimicking interparticle collisions but can model the collision between the particle and fiber (or other particles) when the particle’s center of mass is attached to the fiber. As a result, particle collection efficiency for interception events is inaccurate since the particles are collected on the lateral side of the fiber [19]. This problem has been solved by developing user-defined functions that allow the solver to assume capture when the distance between the particle and the fiber is less than the particle radius [19, 63, 82]. The particle trajectories for all these processes can be calculated using the Lagrangian method where all the Newtonian forces are applied on the particle as it moves through the flow domain [114]. When a particle is captured on the fiber, the amount of the volume occupied by the particle is subtracted from the volume of the cell. Continuous particle deposition leads to the dendrites formation as shown in Fig. 8. As the dendrite’s formation increases, less volume will be available for air to flow as the filter will start to clog and pressure drop across the filter will increase.

### Pressure drop across clogged filters

The total pressure drop across a filter is the sum of pressure drop due to the clean filter $\Delta {P}_{f}$ and the pressure drop due to particle deposition $\Delta {P}_{p}$ as shown in Eq. 21 [115].

21 $$\Delta P = \Delta P_{f} + \Delta P_{p}$$

Alternatively, research proposed the division of a filter into different layers. The total pressure drop was calculated as the sum of pressure drops in each layer during particle deposition inside the filter followed by the dendrites formation up to the surface. These dendrites then act as new fibers to collect the incoming particles. It was explained that the total pressure drop in a filter is the sum of pressure drops in each layer at a given time [116]. The expression for the pressure drop is given as:

22 $$\Delta P_{t} = \sum\nolimits_{j = 0}^{np} {\Delta P_{J,t} }$$

where j represents the layer. For this model, it was considered that filtration is mainly as depth filtration [48]. The pressure drop across the filter keeps on increasing due to particle deposition and dendrite formation. Continuous deposition leads to the total pressure increase with time and reaches a maximum point where the filter is considered fully clogged. The pressure drop in the clogged filter is given by Eq. 23.

23 $$\Delta P = 64\mu V_{0} Z\left( {\frac{{\alpha_{f} }}{{d_{f} }} + \frac{{\alpha_{p} }}{{d_{p} }}} \right) + \left( {\frac{{\alpha_{f} }}{{d_{f}^{2} }} + \frac{{\alpha_{p} }}{{d_{p}^{2} }}} \right)^{1/2}$$

where μ is air velocity, *V*_0_ is the filtration velocity, *Z *is the filter thickness, ${\alpha}_{f}$ is the filter packing density, ${\alpha}_{p}$ is the particle density, and *d*_*p*_ and *d*_*f*_ are particle and fiber diameter. Later, it was found that the above expression gives a low-pressure-drop value for particle size between 0.15 and 0.25 um [117].

The pressure drop from depth filtration to surface filtration is calculated in three stages, i.e., capillary filling, bridging, and cake formation. The connected pores in the filter that allow fluid flows are termed capillaries [112–118]. Particles are first deposited in the capillaries, through depth filtration. When these capillaries are filled up to 40%, the particles start to interact with the particles with neighboring capillaries through a process called bridging. The transition from depth to surface filtration occurs in bridging. As more particles are deposited, these bridges connect and form a cake layer. Now, new particles will be deposited on this cake layer, which is called surface filtration. Equation 24 depicts the pressure drop in the capillary filling phase where ξ (Eq. 26) is the filling coefficient. Here, m is mass of the aerosol deposited, A is filtering cross-sectional area, h is skin layer thickness, $\rho_{s}$ is the aerosol density, Φ0 is the porosity, and $\upvarepsilon _{s}$ is the actual solid volume fraction. The initial pressure drop is given by Eq. 25. Equation 27 shows the time dependency of the radius during deposition.

24 $$\frac{\Delta P}{{\Delta P_{0} }} = \frac{1}{{\left( {1 - \xi } \right)^{2} }};\xi \le \, 0.4$$

25 $$\Delta P_{0} = \frac{8\mu uh}{{\Phi_{0} R_{0}^{2} }}$$

26 $$\xi = \frac{m/A}{{\Phi_{{0\rho_{s} }} \varepsilon_{s} h}}$$

27 $$\frac{R}{{R_{0} }} = \sqrt {1 - \xi }$$

28 $$n = \Phi_{0} \left( {\frac{{R_{f} }}{{R_{0} }}} \right)^{2}$$

The pressure drop in the bridging is given by Eq. 29 while pressure drop in the cake is shown in Eq. 30. The symbols were explained earlier.

29 $$\frac{\Delta p}{\Delta {p}_{o}}=\frac{1}{{\left(1-{\xi}_{i}\right)}^{2}}+{\sum}_{j=1}^{n} \frac{1}{{\left[1+g\left({\xi}_{j+1}-{\xi}_{i}\right)\right]}^{2}} \frac{1}{{\left[1-\left({\xi}_{j+1}-{\xi}_{j}\right)\right]}^{2}}$$

30 $$\Delta P=\Delta Ps+ \mu u\alpha (m/A)$$

### Theoretical analysis

Particle filtration efficiency (PFE) and pressure drop across an air filter mat are often inversely related. If the capture is fully dependent on mechanical entrapment, there is not much room to change this relationship. This is where the application of this mat outperforms many fiber mats described in the literature, particularly in terms of the flow rate or face velocity to which these mats were subjected.

It should be noted that for PM ≤ 1 μm, the inertial impact has little effect because the masses of these particulates are extremely low, making filtering practically impossible unless the pores are much smaller than the particles. The latter condition, on the other hand, has a negative impact on flow. The two principal separation mechanisms are interception, which is dominated by the streamlined flow and flow disruptions imposed by the fibrous architecture, and diffusion, which is governed by the Brownian motion of the particles. The former is more common in 0.5–1 μm particles, whereas the latter is more common in 0.01–0.1 μm particles. As a result, particles with diameters of 0.2–0.3 μm are usually referred to the most penetrating particles (MPPs) [119–121].

The presence of a cylindrical fiber deflects the windward streamlines to the extent of their diameter, as demonstrated in the literature. As a result, larger fibers will naturally displace more incoming fluid, moving particles a greater distance. If the distance between the fiber surface and the particle exceeds the nominal distance required for van der Waals contact, the particle will not “stick” to the fiber, which appears to be the driving force for keeping the particle glued to the fibers. In the absence of any surface charge or chemical reaction, the finer fiber is a superior option for nanoparticle separation as a filtering medium. As a result, nanofibers should be more effective [122, 123]. To evaluate the efficiency in nanofibers, the single-fiber filtration efficiency (SFFE) technique is frequently recommended and given in equation. Payet (1991) presented the SFFE–slip flow relationship:

31 $$\eta_{SFFE} = \eta_{D} + \eta_{R} = 1.6\left( {\frac{1 - \alpha }{{Ku}}} \right)^{1/3} Pe^{^{\prime\prime} - 2/3} + 0.6\left( {\frac{1 - \alpha }{{Ku}}} \right)\left( {\frac{{R^{^{\prime}2} }}{{1 + R^{\prime}}}} \right)$$

where $\eta_{D}$ is the diffusion-based efficiency, $\eta_{R}$ is the interception-based efficiency, α is the fiber packing density, and Ku is the Kuwabara number, which is expressed as:

32 $$Ku = \, - \frac{ln\alpha }{2} - \frac{3}{4} + \alpha - \frac{{\alpha^{2} }}{4}$$

Equations 33, 34 define various other parameters associated with Eqs. 32, 33:

33 $$\left\{ \begin{gathered} Pe^{\prime\prime} = Pe^{\prime}C_{d}^{^{\prime} - 3/2} \hfill \\ Pe^{\prime} = PeC_{d}^{ - 3/2} \hfill \\ C_{d} = 1 + 0.388Kn\left( {\frac{{\left( {1 - \alpha } \right)Pe}}{Ku}} \right)^{1/3} \hfill \\ C^{\prime}_{d} = \frac{1}{{1 + 1.6\left( {\frac{{\left( {1 - \alpha } \right)}}{Ku}} \right)^{1/3} Pe^{ - 2/3} C_{d} }} \hfill \\ \end{gathered} \right\}$$

34 $$\left\{ \begin{gathered} \left( {\frac{{R^{{\prime}{2}} }}{{1 + R^{\prime}}}} \right) = \left( {\frac{{R^{2} }}{1 + R}} \right)C_{r} \hfill \\ R = \frac{{d_{p} }}{{d_{f} }} \hfill \\ C_{r} = 1 + \frac{1.999Kn}{R} \hfill \\ \end{gathered} \right\}$$

where *C*_*d*_ is the slip correction factor and *R *is the interception parameter defined as the ratio of particle diameter and fiber diameter. The total efficiency of the filter mat can be defined using Eq. 35:

35 $$\eta = 1 - \exp \left( { - \frac{{4\alpha \eta_{SFFE} t}}{{\pi \left( {1 - \alpha } \right)d_{f} }}} \right)$$

where t is the nanofiber mat thickness. Considering the diffusion coefficient D ~ 10^–5^ cm^2^/s for 0.2 μm. Some research calculates and compares SFFE efficiency with experimental particle filtration efficiency for validation. Hence, theoretical explanation predicts greater efficiency in nanofiber fibers than microfibers and provides us with a qualitative understanding [119, 120, 124, 125].

## Experimental validation

**Figure 9:**
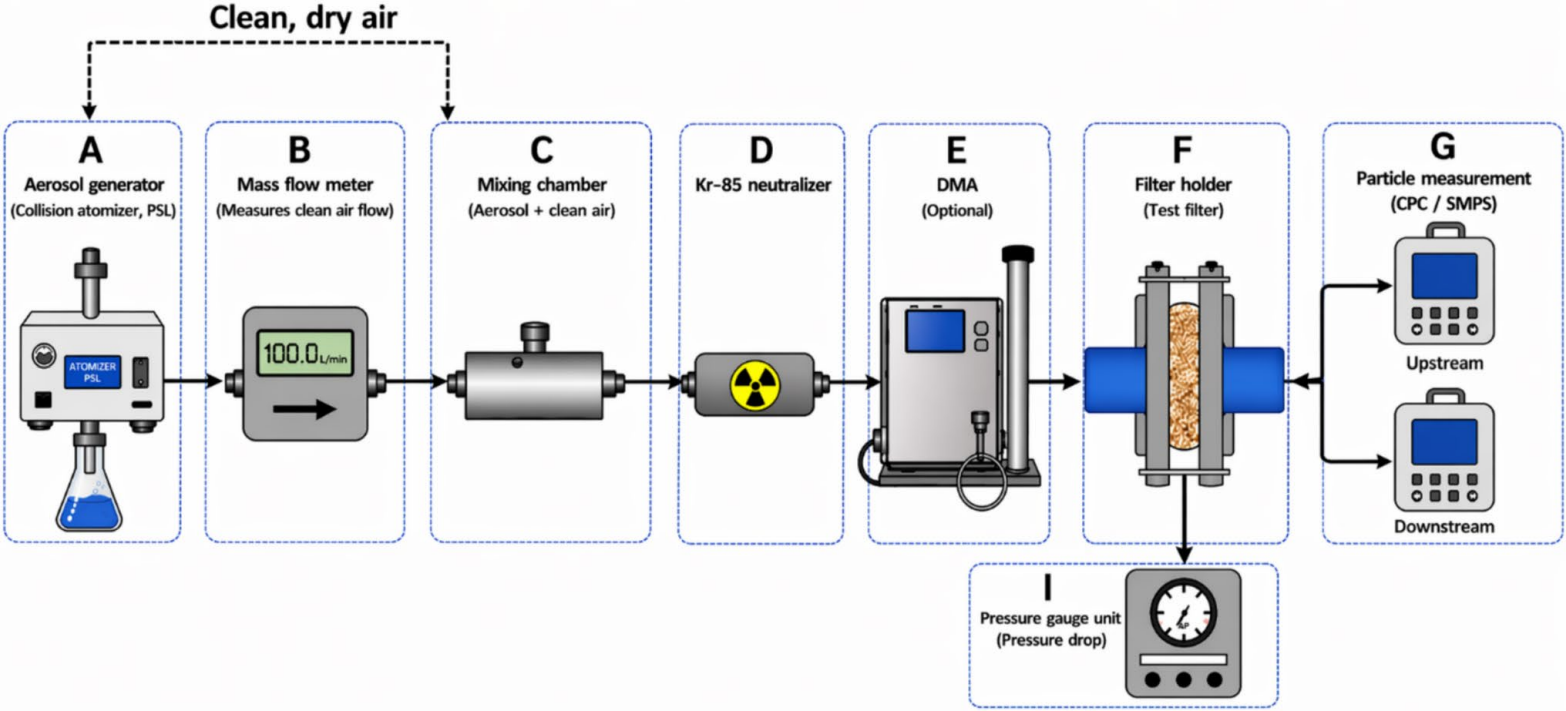
Schematic of the experimental setup for submicron aerosol filtration testing. A collision atomizer **A** is used to generate stable submicron aerosol particles (e.g., PSL or NaCl). The generated aerosol is transported through a controlled flow system, where the volumetric flow rate is monitored using a mass flow meter **B**. The aerosol stream is then introduced into a mixing chamber **C** to achieve uniform particle concentration and stable dispersion. A Kr-85 neutralizer **D** is employed to bring the aerosol to a Boltzmann equilibrium charge distribution. When required, a differential mobility analyzer (DMA) **E** is used to classify particles and generate monodisperse aerosols. The conditioned aerosol is passed through the filter holder **F**, where the test filter medium is mounted. Particle concentrations upstream and downstream of the filter are measured using particle measurement instruments **H**, such as CPC or SMPS, to determine filtration efficiency. The pressure drop across the filter is simultaneously monitored using a differential pressure gauge **I**. This setup represents a standard configuration for evaluating submicron filtration performance of nanofiber and respirator-type filters.

All numerical models must be validated with experiments to establish their accuracy. A suitable test setup should be able to generate aerosols of different characteristics and allow testing the performance of the filters made from different media. A schematic diagram of the proposed experimental setup is shown in Fig. 9

An essential component of the experimental system is the aerosol generation and conditioning unit, as illustrated in Fig. 9. In the present configuration, a collision atomizer (Fig. 9A) employed to generate stable submicron aerosol particles typically composed of polystyrene latex (PSL) or sodium chloride (NaCl). Atomizer-based aerosol generation is widely used in filtration studies due to its ability to produce reproducible particle concentrations within the submicron size range, which is particularly relevant for evaluating nanofiber and respirator-type filter media (NIOSH, 42 CFR Part 84; ISO 16900) [126]. The Aerosol Generator Model 3076 can generate aerosol concentrations greater than 10^7^ particles/cm^3^ while maintaining a nominal flow rate of 3.0 L/min. It supports the production of a range of aerosol types, including polystyrene latex (PSL) particles up to 2 µm in diameter, salt aerosols with modal diameters between 20 and 100 nm, and oil-based aerosols [127].

The generated aerosol is transported through the system under controlled flow conditions, where the mass flow meter (Fig. 9B) is used to monitor and regulate the airflow. The flow meter measures only the clean air stream to avoid aerosol contamination and ensure accurate flow control. The aerosol stream is then introduced into a mixing chamber (Fig. 9C), where it is diluted and homogenized to achieve a stable particle concentration and uniform distribution across the flow domain.

Since aerosol particles generated through atomization processes may carry electrical charges, a Kr-85 neutralizer (Fig. 9D) is employed to bring the particles to a Boltzmann equilibrium charge distribution. Kr-85 neutralizer (Fig. 9D) uses a radioactive source (Kr-85) for neutralization. The source creates charged ions in the atmosphere that neutralize the charged aerosols. This step is essential to minimize electrostatic bias and ensure that the measured filtration performance reflects intrinsic particle capture mechanisms rather than artificially enhanced electrostatic effects. Different bipolar neutralizers have been utilized but Kr-85 (TSI Model 3012) neutralizer has been used previously as it is suitable for use with flows up to 50 L/min [101–103].

For experiments requiring precise particle size control, a differential mobility analyzer (DMA) (Fig. 9E) may be incorporated to classify particles based on their electrical mobility and produce monodisperse aerosols. However, in many filtration studies, polydisperse aerosols are sufficient, and the DMA is therefore considered an optional component depending on the experimental objective. The DMA can classify different particles ranging between 3 and 250 nm. DMA (DMA TSI model 3081) can be used to produce monodisperse aerosol particles [17, 128, 129]. The particles are then neutralized to eliminate the impact of charges on the filter penetration. Following aerosol conditioning, the particle-laden flow is directed to the filter holder (Fig. 9F), where the test filter medium is mounted.

Filtration performance is evaluated by measuring particle concentrations both upstream and downstream of the filter using particle measurement instruments (Fig. 9H), such as condensation particle counters (CPC) or scanning mobility particle sizers (SMPS) or optical particle counter (OPC). These measurements enable the determination of filtration efficiency as a function of particle size. Optical particle counters (OPC) are employed to analyze the aerosol particle size as well as they can count the particles [130]. The other aerosol properties like density, absorption, and shape are not detected by OPC. The lower limit size for particle detection for light scattering OPCs is 0.05–5.0 um upper size limit ranges between 0.2 and 100.0 um [131]. An aerodynamic particle sizer (APS) can be used as an alternative to the optical particle counter. APS can calculate both particle size and concentration. The APSs are more effective in measuring the size of the aerodynamic particles, which have a diameter range between 0.5 and 20.0 um [132]. Scanning mobility particle analyzers (SMPSs) are comparable with APSs. For particles diameter less than 30.0 um, SMPS overestimates the mass concentration of the monodisperse particles. For polydisperse particle size between 0.09 and 0.29 um, SMPS overestimates mass concentration [133, 134]. Condensation particle counter (CPC), also known as condensation nucleus counter (CNC), is an addition to the OPCs, which are used to increase their particle detection range. The particles that are very small and are difficult to detect are passed through a chamber with a condensable fluid. The supersaturated vapors of the liquid condense on particles to grow the size which can be detected by OPC [130]. For example, CPC (TSI Model 3775) can detect particles down to 4 nm.

Controlling particle injections during any experiment is critical to obtaining accurate results. This is often due to a multimodal distribution of particle sizes. This means that the aerosols could have a major concentration of particles in a narrow particle size range or a few such ranges. The absolute particle count could differ by a few orders of magnitude. Therefore, knowing the size, concentration by count, or weight is the prerequisite. Several aerosol spectrometers detect, count, and size particles using laser scattering and yield best results when the particles are adequately spaced in the sensing volume. Therefore, it is critical to ensure that the particle concentration in the volume does not exceed prescribed limits to eliminate several particles coming together and being counted falsely as one big particle [135–137]. For example, TSI OPS 3300 prescribes a maximum particle count of 3,000/cm^3^ [138]. Coincidence errors could be prevalent in a certain class of test dust particles in which the count of smaller particles could be several orders of magnitude compared to larger particles [139]. Controlling the particle injection rate in the flow domain and monitoring the instantaneous concentrations in all the channels of the spectrometer significantly alleviate this critical source of error in particle measurement.

Simultaneously, the pressure drop across the filter is measured using a differential pressure gauge (Fig. 9I), which is critical for evaluating airflow resistance and overall filter performance. The combined analysis of particle concentration and pressure drop provides a comprehensive assessment of filtration behavior.

It should be noted that the experimental configuration presented in Fig. 9 is specifically designed for submicron aerosol filtration testing, which is commonly used in respirator and nanofiber filter evaluation (NIOSH, 42 CFR Part 84). Aerosol generation and testing methodologies vary significantly depending on the filter application, particle size range, and relevant standards. For example, while atomizer-based systems are typically used for submicron respirator testing, dry dust injectors are commonly employed for engine intake and HVAC filter evaluations involving larger particles, as specified in standards such as ISO 5011 and ISO 16890. A comparative overview of different testing configurations is provided in Table 1.

**Table 1:** Comparison of aerosol generation, conditioning, and measurement configurations used in filtration testing for respirator, engine intake, and HVAC applications

Parameter	Respirator testing (submicron)	Engine intake filters (coarse dust)	HVAC filters (broad range)
Typical application	Personal protective equipment (e.g., N95 respirators)	Automotive engine air intake systems	Building ventilation and air conditioning systems
Relevant standards	NIOSH 42 CFR Part 84, ISO 16900	ISO 5011	ISO 16890
Particle size range	~ 0.02–0.6 μm (focus on MPPS ~ 0.3 μm)	~ 0.5–100 μm (polydisperse coarse dust)	~ 0.3–10 μm (PM1, PM2.5, PM10 classification ranges)
Aerosol generator type	Collision atomizer (NaCl, PSL aerosols)	Dry dust feeder/ISO dust injector	Atomizer or dust feeder (depending on test class)
Aerosol type	Liquid-generated, dried aerosol (mono- or polydisperse)	Dry, polydisperse test dust (e.g., ISO A2 fine dust)	Liquid aerosol (fine fraction) or dry dust
Need for diffusion dryer	Not typically required in standard tests; may be used in research setups	Not required (dry particles)	Optional (depends on aerosol type)
Pre-classification (cyclone/impactor)	Not used in standard respirator test protocols	Not typically used in standard testing	Rare; depends on experimental setup
Charge neutralization	Typically required (e.g., Kr-85 neutralizer)	Not typically required	Optional (mainly in research setups)
Particle size selection (DMA)	Often used (for monodisperse testing)	Not used	Rarely used (mainly research applications)
Mixing/dilution system	Required (to control concentration and flow stability)	Required (to ensure uniform dust dispersion)	Required (to stabilize aerosol concentration)
Measurement technique	CPC/SMPS (number-based, size-resolved)	Gravimetric methods, arrestance, dust-holding capacity	Optical particle counters (OPC), fractional PM efficiency
Filtration metrics	Efficiency vs particle size, MPPS, pressure drop	Arrestance, dust-holding capacity, pressure drop	Fractional efficiency (PM1, PM2.5, PM10), pressure drop
Flow conditions	Controlled low flow (standardized face velocity)	High flow (engine operating conditions)	Moderate flow (ventilation conditions)
System complexity	High (precise aerosol conditioning and sizing)	Moderate (robust dust feeding system)	Moderate (depends on classification level)

The selection of aerosol generator, particle size range, and measurement technique is strongly dependent on the intended application and relevant testing standards, including NIOSH 42 CFR Part 84, ISO 5011, and ISO 16890. The table highlights key differences in aerosol characteristics, instrumentation, and performance metrics across these filtration systems

## Fiber manufacturing techniques

### Electrospinning

**Figure 10:**
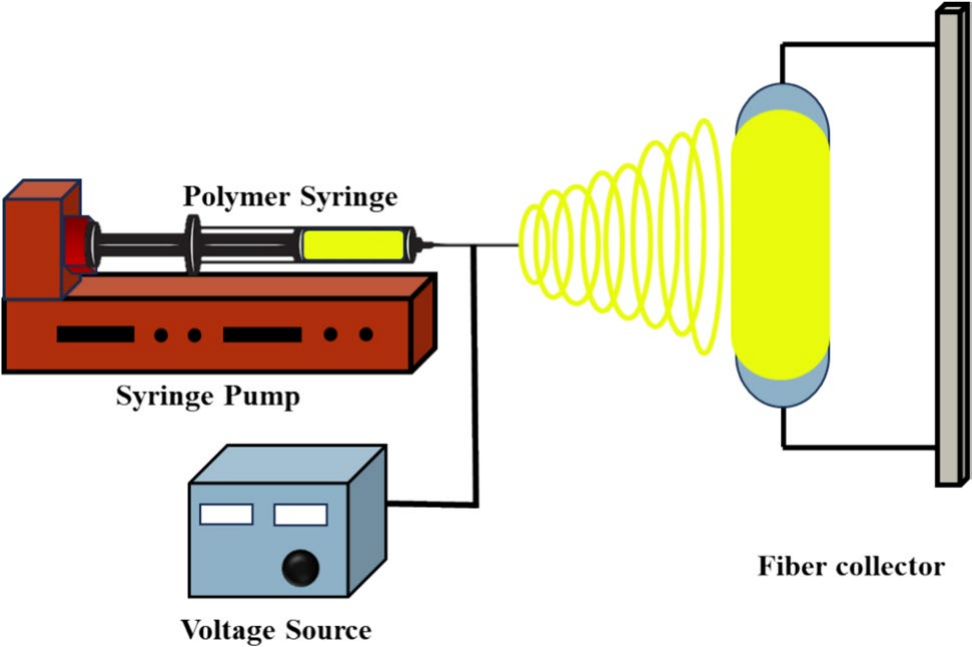
Electrospinning setup for nanofiber production.

The process of electrospinning harnesses electrostatic forces to transform polymer solutions into fine fibers, reducing their diameter from millimeters to micrometer or nanometer scales. A typical electrospinning setup comprises a syringe pump equipped with a metallic needle, a high voltage source, and a grounded collector as shown in Fig. 10. The syringe pump regulates the flow rate of the polymer solution, ensuring optimal conditions to prevent fiber wastage or breakage. Upon application of voltage, electrostatic repulsion forces dominate, causing the solution to droplet at the needle tip to deform into a characteristic Taylor cone. When electrostatic forces overcome surface tension, a fine jet of polymer is ejected, undergoing stretching and whipping motions induced by the electric field. This whipping motion aids in solvent evaporation, leading to the solidification of the elongated filament onto the collector surface. The collector, which may be rotating or stationary, determines the alignment of the deposited fibers. Additionally, the diameter of the metallic needle influences the flow rate and applied voltage. Proper grounding of the collector ensures uninterrupted fiber deposition. Various parameters, including temperature, heating or cooling rate, and holding time during calcination, further influence the electrospinning process [140, 141].

Electrospinning (ES) has been recognized for its ability in producing high-efficiency nanofibers but is faced with significant production efficiency challenges. Traditional single-needle electrospinning systems exhibit low mass yield, typically in the range of 10 −100 mg/min (equating to about 0.6 − 6 g/h), indicating that they are energetically intensive for the quantity of nanofibers generated [142]. This has led to the characterization of electrospinning as “economically unfeasible due to high energy consumption” at scale, as the cumulative energy per unit mass of nanofibers tends to be high despite each fiber consuming relatively little energy during formation. Studies confirm that conventional systems exhibit production rates significantly lower than alternative techniques such as meltblowing, which can yield production rates reaching up to 1500−1800 tons/year [142, 143]. Efforts to enhance the scalibility have led to various innovations, including multi-needle setups and needleless electrospinning (e.g., using rotating drums). Some of these approaches have resulted in increased throughput, with multi-needle systems achieving production rates reaching up to 0.618–0.712 g/h, but still insufficient for large-scale applications [144–146]. Needleless electrospinning has shown potential for mass production, with reported production rates of 3.1 – 8.6 g/h depending on the apparatus used [147]. Despite these advancements, the current state of industrial electrospinning technology still produces outputs that are several orders of magnitude lower than what is achievable with meltblowing processes [148].

Electrospun nanofiber membranes achieve high filtration efficiency primarily due to their submicron fiber diameters, which enhance diffusion- and interception-dominated particle capture near the most penetrating particle size (MPPS). Due to their high porosity and fine fiber diameters, these membranes significantly enhance particle adhesion capabilities, particularly for nanoscale particles that adhere primarily through diffusion. There is evidence that reducing fiber diameter can improve filtration efficiency, especially for the most penetrating particle sizes (MPPS), which range from hundreds of nanometers down to tens of nanometers[149, 150]. Research has indicated that nanofibers with diameters around 60 nm can achieve filtration efficiencies of approximately 85–91% for submicron particles, particularly in single-layer membranes [151]. Another study reported a filtration efficiency of 99.7% for a particle size of 100 nm [152].

Electrospinning (ES) is a versatile method that can turn many polymers, composites, and ceramics into nanofibers. This flexibility enables the use of high-performance materials that are particularly relevant for demanding environments such as mining, where factors like thermal stability, chemical resistance, and flame retardancy are crucial. For instance, polymers such as polyacrylonitrile (PAN), polyvinylidene fluoride (PVDF), and polyamides are readily electrospinnable and possess mechanical, thermal, and chemical stability suitable for filtration under harsh operating conditions. [153].

Materials such as PVDF offer inherent hydrophobicity, providing stable filtration performance in moist environments. In addition, PVDF exhibits good thermal and chemical resistance, making it suitable for mining environments that involve elevated temperatures and reactive gases. PVDF electrospun nanofibers resist humidity, preventing filter collapse and biological growth. The various feasible polymer choices facilitate the design of electrospun structures that can withstand mechanical stresses and harsh operating conditions commonly encountered in mining environments [153, 154]. Moreover, electrospinning can be applied to ceramics and composite precursors, expanding its utility beyond traditional organic polymers [155]. For instance, using electrospun ceramic fibers enhances material toughness and allows for the production of robust composites suitable for industrial applications, including filtration in mines [156]. This novel capability is vital when considering advanced hybrid materials that integrate multiple functionalities, anti-static and flame-retardant fibers [157]. Electrospinning is limited by solvent availability and low conductivity and, in some cases, by the need for high-temperature post-processing [158, 159]. Despite these constraints, its broad material compatibility enables the fabrication of functional filaments tailored to diverse mining applications [160].

**Figure 11:**
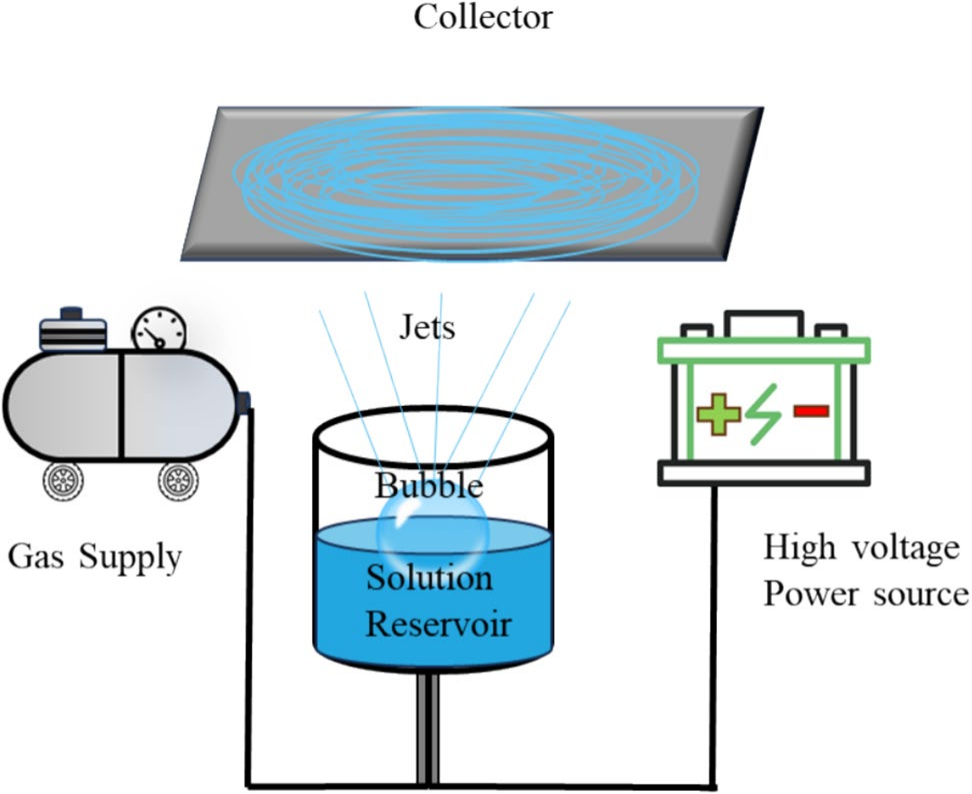
A typical bubble electrospinning setup.

Bubble electrospinning enables higher nanofiber throughput than single-needle electrospinning by generating multiple simultaneous jets from electrically charged bubble surfaces. In the BE process, an air bubble is induced to emerge at the surface of a polymer solution, which is positioned within a high electric field as shown in Fig. 11. The application of high voltage causes charge transfer from the vessel to the polymer solution covering the surface of the bubble. Upon reaching the threshold voltage, multiple jets of nanofiber emanate from the surface of the bubbles, directed toward a grounded collector. By increasing the quantity of air bubbles present at the polymer solution’s surface, a significant number of jets can be simultaneously ejected, facilitating the large-scale production of nanofibers [123, 124]. BE uses slightly lower voltage (6–8 kv) compared to ES (10 kv).

Bubble electrospinning enables the formation of ultrafine nanofibers with diameters well below those typically achieved by needle-based electrospinning, resulting in enhanced particle capture efficiency. A critical dimension to understand is the filtration deficiency in bubble electrospun fibers, which appears to be influenced by various parameters including fiber diameter, solution concentration, and morphology.

Bubble electrospinning demonstrates substantially higher production efficiency than conventional needle-based electrospinning by enabling simultaneous multi-jet fiber formation [161, 162]. This novel approach leverages the unique properties of bubbles, whereby their surfaces are charged in an electric field, leading to the efficient formation and ejection of nanofibers as the bubbles burst [163, 164]. This mechanism not only increases the rate of fiber formation but also facilitates the generation of finer, more uniform nanofibers with superior properties [165]. In quantitative terms, bubble electrospinning has been reported to yield production rates of up to 16.9 g/h, markedly higher than the 0.16 g/h typically achieved with needle systems [166]. The filtration efficiency for NaCl aerosol for a median diameter of 75 nm is reported to be 94% [167].

In BE, any polymer that can form stable bubbles in a solution while carrying an electric charge can theoretically be employed, offering a diverse selection of materials [168]. A significant advantage of this method is the ability to utilize aqueous polymer solutions, thereby alleviating health and environmental concerns associated with toxic organic solvents [169]. For example, polyvinyl alcohol (PVA) and other water-soluble polymers can be effectively processed through BE, making them suitable options for biodegradable and eco-friendly filter media, particularly valuable in sustainability-focused applications [170]. BE enables low-temperature processing of heat-sensitive polymers such as polyimides, which degrade during melting processing. This characteristic enhances the versatility of BE for designing fibers intended for high-performance applications [171].

### Meltblowing

Meltblowing is an industry-standard process for large-scale production of nonwoven filter fabrics due to its high throughput and compatibility with existing personal protective equipment (PPE) manufacturing systems. Meltblowing produces microfibers with diameters typically in the 1–10 μm range, with polypropylene fibers averaging around 2 μm under standard processing conditions[172]. The resulting fiber diameter distribution is relatively polydisperse compared to electrospinning, yielding fibers one to two orders of magnitude larger than those produced by electrospinning or supersonic solution blowing. Consequently, meltblown nonwovens exhibit larger pore sizes and lower specific surface area, making them effective for capturing respirable dust particles (e.g., > 1 μm) but less efficient for ultrafine particles when relying solely on mechanical filtration mechanisms [173].

Meltblowing is constrained in material compatibility relative to solution-based fiber fabrication methods. The process is limited to thermoplastic polymers that can be melted and extruded, making polypropylene (PP) the dominant material due to its low melting temperature (~ 165 °C), favorable melt flow characteristics, and inherent hydrophobicity. Other polymers, such as polyesters (PET), can be processed by meltblowing but are generally more difficult to handle and less commonly adopted in industrial practice [173]. In addition, meltblowing is not suitable for non-thermoplastic materials or polymers that decompose prior to melting, including most natural polymers and high-temperature polymers such as PTFE and aramids [174, 175]. Despite their relatively coarse fiber diameters, meltblown polypropylene filters can achieve high initial filtration efficiencies due to the presence of electret charges embedded within the fibers. Meltblown PP filters have demonstrated filtration efficiencies exceeding 95% for particles around 300 nm, a size range associated with minimum mechanical capture efficiency [176]. The electrostatic charges on the fibers enhance particle capture by attracting particles that would otherwise penetrate the filter, allowing meltblown media to outperform uncharged materials such as cotton or cellulose filters with similar pore sizes [177]. This electrostatic enhancement is particularly advantageous in underground mining environments, where effective removal of nanoscale dust and diesel particulate matter is required during initial filter use. However, environmental factors such as high humidity, exposure to oils, or cleaning procedures can dissipate these electret charges, leading to reduced filtration performance for ultrafine particles. For example, exposure of meltblown N95 filters to 75% ethanol has been shown to reduce filtration efficiency below 95% due to charge neutralization [178]. Similarly, prolonged exposure to moist conditions can gradually degrade electrostatic charge, diminishing nanoparticle capture efficiency over time[179].

### Supersonic solution blowing

Supersonic solution blowing (SSB) is a technique that combines electrostatic fiber formation with intense aerodynamic stretching to produce ultrafine nanofibers, typically in the 20–50 nm diameter range. In SSB, a polymer solution is expelled into a supersonic airflow generated by a Laval nozzle while subjected to an electric field. Experiments have reported nanofibers as thin as ~ 20–50 nm for polymers such as nylon 6 and polyacrylonitrile, whereas conventional electrospinning under similar conditions typically produces fibers in the ~ 200–500 nm range. The supersonic airflow imposes an extremely high shear rate (on the order of 10^10^ s⁻^1^) on the solution jet, rapidly stretching and thinning the fibers while promoting fast solvent evaporation. In addition to enabling extreme fiber thinning, SSB exhibits broader material and solvent compatibility than conventional electrospinning, as the strong aerodynamic forces reduce reliance on highly volatile or toxic solvents and allow processing from benign solvents such as water or ethanol [180].

The ability of SSB to consistently produce sub-100-nm fibers is particularly advantageous for nanoparticle filtration, as fibers in this size regime generate strong inter-fiber van der Waals interactions that significantly enhance particle capture. Theoretical analysis confirms that fibers below ~ 100 nm diameter produce attractive force fields capable of drawing in nanoparticles within ~ 100 nm of the fiber surface, thereby substantially increasing capture efficiency for ultrafine particles [28].

SSB-derived nanofibers have demonstrated superior filtration performance compared to conventional electrospun fibers due to their substantially smaller diameters. Filters coated with a thin layer of 20–50-nm SSB nanofibers exhibited enhanced capture of aerosolized nanoparticles relative to uncoated media, as reduced fiber diameters improve interception and diffusion-dominated filtration mechanisms. Furthermore, SSB nanofiber layers outperform electrospun fiber layers with larger diameters (~ 300 nm), confirming the effectiveness of finer fibers for nanoparticle capture [181]. Studies on SSB nanofiber mats fabricated from recycled PET for facemask applications reported filtration efficiencies exceeding 99% for PM2.5, which were maintained after 21 days of continuous use with minimal performance degradation. These mats also demonstrated high durability, retaining > 99% efficiency after 10 hand-wash cycles, indicating suitability for environments such as underground mines where prolonged use and exposure to humidity are common [180, 182, 183].

SSB has also been shown to enable nanofiber formation from polymers that are difficult or impossible to process by conventional electrospinning. Natural polymers, biopolymers, and materials such as natural rubber latex have been successfully processed using SSB, as the supersonic airflow overcomes cohesive forces that hinder electrospinning. This expanded solvent and polymer compatibility allow SSB to produce nanofibers from mechanically robust, mine-suitable polymers such as polyamides and polyesters, which exhibit greater resistance to humid and abrasive conditions than ultrafine polypropylene fibers [170, 175–180].

### Material, process, and performance relationships in fibrous filter manufacturing

Fiber fabrication techniques govern not only fiber morphology but also the electrostatic state of fibers, charge retention behavior, and ultimately filtration performance. These relationships are inherently process-dependent because each fabrication route imposes distinct mechanical, thermal, and electrical fields during fiber formation. As a result, filtration efficiency cannot be explained solely by fiber diameter. Instead, performance emerges from a coupled interaction between structure, material chemistry, and electrostatic behavior. Table 2 summarizes the key performance characteristics of the four major fabrication routes discussed in this section.

**Table 2:** Comparative summary of fibrous filter fabrication method

Parameter	Electrospinning	Bubble electrospinning	Meltblowing	Supersonic solution blowing
Fiber diameter range	Ultrafine fibers, typically nanoscale to low microscale (300 nm–few μm) [78, 119, 184, 185]	Nanoscale fibers; specific reported values around 90–150 nm in one study, but strongly process-dependent [186]	Micron-scale fibers, typically around 1–10 μm [187]	Ultrafine fibers (< 100 nm) [152, 188]
Throughput	Low for conventional single-needle electrospinning (10–100 mg/min) [184, 189]	Higher than conventional ES [190, 191]	Very high/industrial scale [192]	Higher than conventional electrospinning; scalable, though industrial adoption is still developing
Charge mechanism	In situ charge generation and trapping during fiber formation [193]	Not yet clearly established	Post-process electret charging, commonly corona-based [194]	Electrostatic + aerodynamic stretching [195]
Charge stability	Moderate to high (depending on material and trap depth) [52, 53, 55, 193]	Limited data/insufficiently characterized	Low to moderate; strongly sensitive to humidity and temperature [194]	Limited data/insufficiently characterized
Material compatibility	Broad (PVDF, PET, PCL) [157, 185]	Broad (PVDF, PET, PCL, biopolymers)[196]	Primarily melt-processable thermoplastics; also supports PLA, composites, and additives [197]	Broad including biopolymers
Filtration efficiency	Can reach very high efficiency for 0.3–2.5 μm (up to 99%) [78, 119, 152]	Up to ~ 94% in a needleless/related free-surface study under NaCl aerosol testing (median diameter 75 nm) [167]	72% at 0.3 μm without electret treatment in one porous PLA system [187] > 95% in electret/composite systems) [194]	Very high, including 99.7% at PM0.1 and 99.99% at PM2 in one study [152]
Key limitation	Low throughput/scalability limits	Charge characterization gap	Environmental charge decay	Limited industrial adoption; charge characterization gap

Electrospinning produces ultrafine fibers ranging from the nanoscale to low microscale, with high surface area and dense fiber networks [159, 191, 198]. The high electric field applied during spinning generates residual charges that become trapped on the fiber surface and within the fiber bulk [198]. These trapped charges create local electric fields that enhance particle capture through induced-dipole interactions, even when particles are electrically neutral [58]. The presence of deep and shallow charge traps further governs charge stability [52, 53, 55]. However, despite these advantages, electrospinning remains limited by low throughput and challenges in scaling conventional single-needle systems [166, 197].

Bubble electrospinning and related needleless approaches attempt to overcome these limitations by generating multiple jets from bubble surfaces. These methods enable higher production rates compared to conventional electrospinning and eliminate issues such as needle clogging [174, 177]. Fiber formation in bubble electrospinning is governed by bubble rupture dynamics and non-uniform electric field distribution, which leads to variability in fiber diameter and morphology [178]. Although these processes demonstrate the ability to produce nanoscale fibers, the electrostatic characteristics of bubble electrospun fibers remain insufficiently understood. In particular, charge generation, trapping, and stability have not been systematically quantified, representing a significant gap in current research.

Meltblowing represents a fundamentally different fabrication route based on high-temperature melt extrusion and aerodynamic attenuation. This process produces fibers in the micron-scale range, typically around 1–10 µm [185], resulting in lower intrinsic surface area compared to electrospun systems. Consequently, mechanical filtration alone is less effective for submicron particles. To compensate, meltblown filters rely on post-process electret charging, commonly through corona discharge, to introduce electrostatic capture mechanisms [152]. These charges are typically stored in shallow traps and are sensitive to environmental conditions such as humidity and temperature, leading to performance degradation over time [63]. While meltblowing enables very high production rates and industrial scalability [199], its dependence on external charging remains a critical limitation.

Supersonic solution blowing provides a hybrid fabrication route that combines strong aerodynamic forces with electrostatic effects. The process subjects polymer jets to extremely high strain rates, leading to significant fiber thinning and the formation of ultrafine fibers in the nanoscale to submicron range [119, 120]. The rapid stretching and deformation induce molecular alignment and promote the formation of electroactive phases, which enhances dipole density and intrinsic electrostatic behavior [155]. In addition, the resulting fiber networks exhibit high porosity and operate in slip or transition flow regimes, which reduces aerodynamic drag and enhances particle capture efficiency [182]. These combined effects allow supersonic solution-blown filters to achieve high filtration efficiencies, including values exceeding 99% for submicron particles, while maintaining relatively low pressure drop [182]. Despite these advantages, large-scale implementation and systematic characterization of charge stability remain limited.

Across all fabrication methods, filtration performance is governed by coupled material–process–electrostatic interactions. Fiber diameter and porosity define airflow pathways and mechanical capture mechanisms, while polymer chemistry and processing history influence charge generation, trap depth, and charge distribution. Interfacial polarization, dipole alignment, and localized electric fields further enhance particle capture, particularly in the submicron regime. These multiscale effects demonstrate that filtration performance cannot be fully understood through geometric parameters alone and that electrostatic phenomena must be considered as an intrinsic component of filter design.

Despite significant progress, several challenges remain. Many studies continue to evaluate filtration performance primarily in terms of fiber size and porosity, while electrostatic charge distribution and stability are often inferred rather than directly measured. The lack of standardized methods for quantifying surface potential and charge decay limits direct comparison across fabrication techniques. In addition, scalability remains a critical barrier for nanofiber-based systems, particularly for electrospinning and emerging techniques such as bubble electrospinning and supersonic solution blowing [183, 197]. Finally, the long-term durability of electrostatic effects under realistic environmental conditions, including humidity, temperature, and particle loading, remains insufficiently understood across different fabrication routes [200].

Addressing these gaps requires a systematic framework that integrates material selection, processing conditions, and electrostatic characterization with filtration performance. Future work should focus on direct measurement of surface potential distributions, identification of charge trapping mechanisms, and controlled studies of environmental charge decay. Such efforts are essential for developing next-generation fibrous filters that combine high efficiency, durability, and scalability.

## Impact factors

### Characteristics of fibrous medium

Fibrous filters are widely employed in aerosol filtration due to their ability to achieve high collection efficiencies across a broad particle size range, particularly for ultrafine and nanoscale particles. The filtration performance of fibrous media is governed by a combination of structural parameters, including fiber diameter, fiber diameter distribution, fiber orientation, and pleat geometry, each of which influences particle capture mechanisms and pressure drop behavior.

Fiber diameter is one of the most influential parameters controlling filtration efficiency. Numerous experimental studies have consistently demonstrated that a reduction in fiber diameter leads to a substantial increase in particle capture efficiency, particularly for ultrafine particles dominated by diffusion and interception [197, 201–203]. This effect arises from the increased surface area per unit mass, reduced pore size, and enhanced probability of particle–fiber interactions. For example, a researcher investigated particles in the size range of 0.03–0.30 μm using fibers with diameters of 100, 201, and 306 nm, reporting collection efficiencies of 99, 88, and 64%, respectively. Similar trends have been observed across a wide range of operating conditions and particle sizes. In another study, fibers with diameters between 520 and 2100 nm were evaluated for 300 nm particles at a flow rate of 13.0 L min⁻^1^, where filtration efficiency decreased monotonically from 85 to 50% as fiber diameter increased [182, 204]. These results were found to persist at higher flow rates, confirming that fiber diameter remains a dominant design parameter across realistic operating regimes.

Beyond single-value fiber diameters, fiber diameter distribution plays a critical role in determining overall filter performance. Unimodal fiber distributions, where all fibers possess a similar diameter, generally exhibit increasing filtration efficiency with decreasing fiber size. In contrast, bimodal distributions, combining coarse and fine fibers, can achieve comparable or enhanced performance when a sufficiently large contrast exists between the two fiber populations [180]. This behavior reflects the ability of fine fibers to capture ultrafine particles while coarse fibers contribute mechanical support and reduced pressure drop. The effectiveness of such mixed architectures has also been demonstrated in PM2.5 filtration studies, where smaller fiber diameters promoted the formation of denser fibrous networks that inhibited particle penetration [125]. Similar conclusions were reported for polyester-based filters with fiber diameters ranging from 17 to 33 μm, where the smallest fiber diameter yielded the highest filtration efficiency despite comparable material composition [183].

Fiber orientation within the filter matrix further modulates filtration efficiency and pressure drop**.** Fibrous media may be broadly classified as unidirectional, random layered, or three-dimensionally random structures [30, 103, 205]. Experimental studies indicate that unidirectional and layered structures often outperform fully three-dimensional random structures for particles in the 1–3 μm size range, owing to more favorable flow paths and increased interception probability[206]. However, other investigations have reported that for random layered structures, particle collection efficiency may be relatively insensitive to in-plane fiber orientation, whereas increased fiber alignment in the perpendicular direction can reduce efficiency for particles between 0.3 and 5.0 μm [12, 199]. These findings highlight the complex interplay between fiber alignment, flow field development, and particle transport mechanisms.

Macroscopic filter geometry, particularly pleat design, exerts a strong influence on pressure drop and dust-holding capacity. Increasing pleat count effectively increases filtration area, resulting in lower pressure drop and enhanced dust loading capability. Studies comparing pleat densities have shown that approximately 15 pleats per inch can minimize pressure drop for particle sizes in the 3–10 μm range [17]. Dust loading experiments further demonstrated that increasing pleat count from 70 to 90 significantly improved dust-holding capacity in several filter configurations, although the magnitude of improvement depended on media thickness and basis weight [198]. In addition to pleat density, pleat shape is also important; triangular or V-shaped pleats have been shown to capture high-velocity particles more effectively than rectangular designs [17, 200, 207]. The pleat design index (*α*), defined as the ratio of pleat height to pleat pitch, has been identified as a critical parameter, with optimal performance reported for α values between 1.48 and 1.59 across a wide particle size range [208, 209].

### Influence of operating conditions

In addition to intrinsic media properties, filtration performance is strongly affected by operating conditions, including particle mass concentration, particle size, relative humidity, and face velocity. These parameters influence particle transport, deposition behavior, and filter loading dynamics. Particle mass concentration significantly affects pressure drop evolution and filtration behavior. Experimental studies have shown that increasing particle concentration can reduce the slope of pressure drop increase during filtration, due to changes in particle deposition patterns [2] At low concentrations, particles tend to form dense, low-permeability cakes, leading to rapid pressure drop increases. At higher concentrations, reduced residence time and spatial constraints promote the formation of looser, more permeable cakes. Similar behavior has been observed in diesel particulate filters, where initial particle deposition within the media causes a sharp pressure rise, followed by linear pressure increase as surface cake filtration becomes dominant [210]. These trends have been quantitatively linked to collected particle mass, with cake formation typically initiating near 1.0 g m^−2^ and governing subsequent pressure drop behavior [211].

Fiber diameter effects persist under varying operating conditions, particularly in the nanoscale regime. Filters composed of fibers with diameters of 100, 201, and 306 nm exhibited collection efficiencies of 99.99, 88, and 64%, respectively, for particles between 30 and 300 nm. Similar enhancements have been reported for polyvinyl acetate nanofiber filters, which achieved efficiencies exceeding 98% for particles as small as 100 nm [201]. These results underscore the dominant role of diffusion-driven capture for sub-100-nm particles, particularly at moderate face velocities [206].

The influence of humidity on filtration performance remains complex and system dependent. Some studies have reported negligible effects of humidity on filtration efficiency for particles smaller than 100 nm over a wide humidity range [212]. In contrast, other investigations observed improved filtration efficiency at elevated humidity levels [213], while additional studies reported performance degradation when relative humidity exceeded 90%, particularly during liquid aerosol [214]. These conflicting observations suggest that humidity effects depend strongly on filter material, particle phase, and loading state.

Face velocity directly influences both filtration efficiency and pressure drop. Linear relationships between pressure drop and face velocity have been demonstrated across multiple filter types [215, 216]. However, filtration efficiency does not always increase monotonically with velocity. Comparative studies of electrospun and supersonically blown filters showed increased efficiency at higher flow rates [119], whereas another investigation reported reduced capture efficiency at lower face velocities for nanoparticles, attributed to changes in Brownian diffusion dominance [217]. These findings highlight the need for careful optimization of operating velocity based on target particle size and dominant capture mechanisms.

### Theoretical analysis

**Figure 12:**
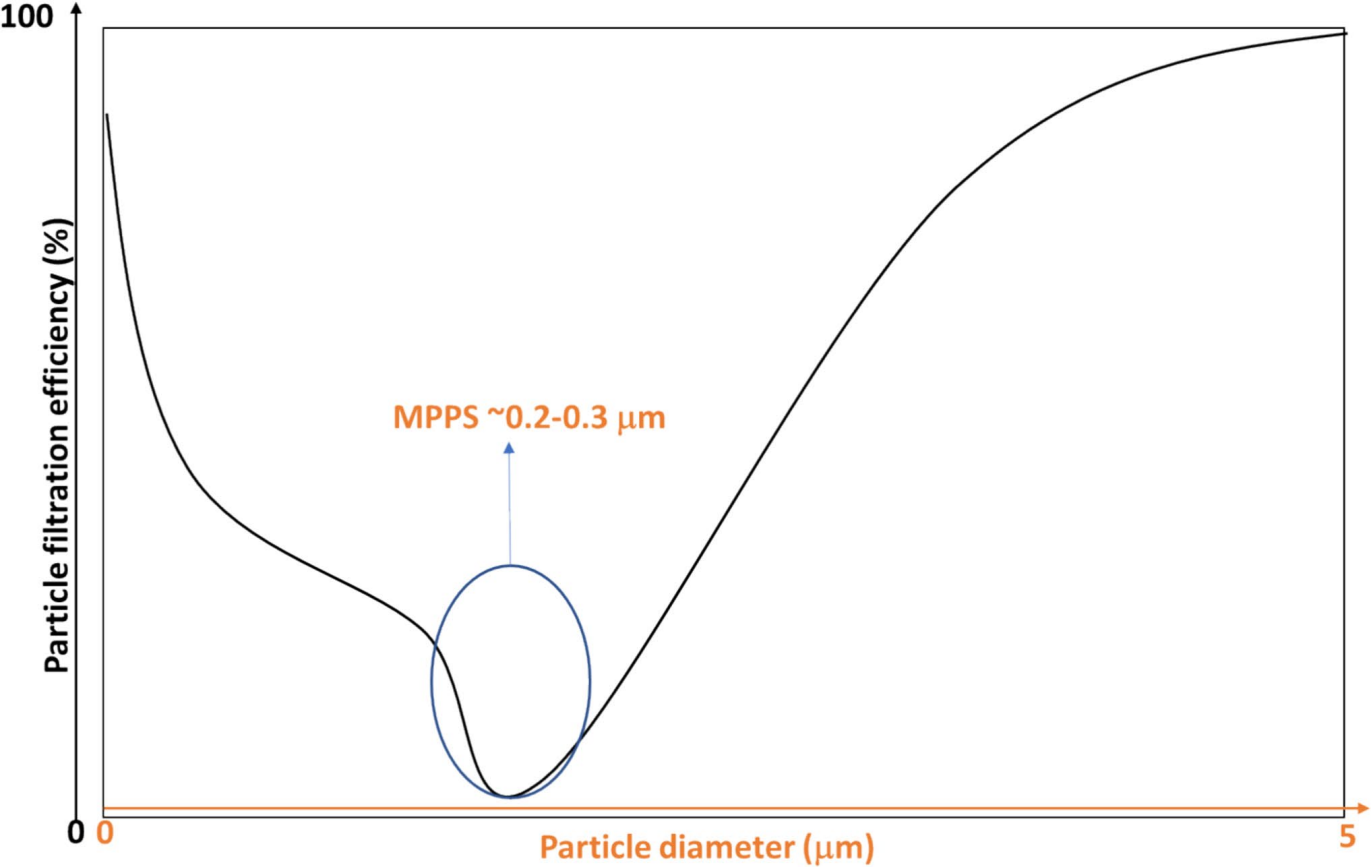
Effect of most penetrating particle size on filtration efficiency.

The term “most penetrating particle size” refers to the particle diameter at which a filter exhibits its lowest filtration efficiency. This phenomenon is intricately tied to the interplay of various capturing mechanisms, including diffusion, interception, and inertial impaction. Figure 12 shows that the particles smaller than 0.1 um exhibit enhanced diffusion as a predominant capturing mechanism. These particles, due to their extremely small size, are subject to Brownian motion induced by thermal energy. Particles smaller or equal to 0.1 µm have a higher probability of being captured due to this diffusion behavior [218]. In contrast, for particles around the MPP size of 0.3 µm, they are relatively larger and have less pronounced Brownian motion. Consequently, diffusion is less effective for these particles. Interception is more effective for particles larger than the MPPS, where the particle’s path intersects the filter fibers. However, for MPPs like 0.3 µm particles, the probability of interception is relatively low, as these particles are smaller and can more easily navigate through the voids between filter fibers. This limited interception contributes to the notion of 0.3-µm particles being the most penetrating. Inertial impaction is most efficient for particles larger than the MPPS. These particles possess sufficient momentum to deviate from the air streamlines and impact the filter media. However, MPPs lack the mass or inertia to follow such a trajectory effectively, allowing them to pass through the filter with limited impact. Figure 12 shows the trend. This PFE will be more effective for particle sizes smaller than 100 nm and larger than 10 nm. The range of 200–300 nm is MPPS. PFE decreases dramatically first until MPPS and then grows as particle size increases. This is since smaller particle sizes, such as 10–100 nm, have more substantial diffusion capturing, but particle sizes larger than MPPS have more inertial impact. As a result, the PFE is lower [119, 120].

**Figure 13:**
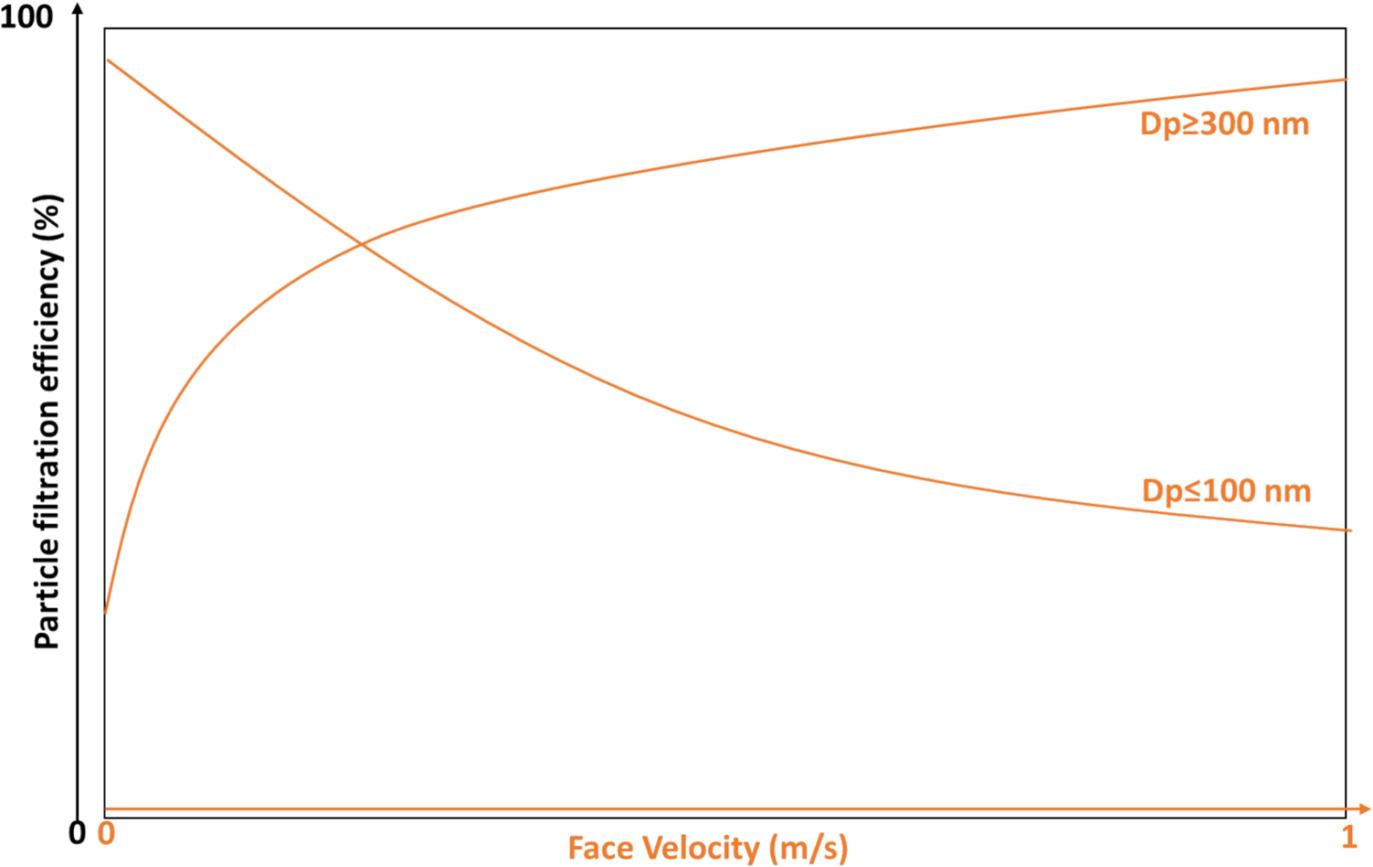
Effect of face velocity on filtration efficiency.

When the particle diameter is fixed, the influence of face velocity may be observed in Fig. 13 PFE increases with larger particle size, i.e., 2.5 μm, as the predominant capture is inertial impact and there are very few chances for the particle to escape with the streamline. However, in the event of MPPS or less, the possibilities of particles leaving with the stream rise since they are lighter in weight and have a high probability of moving out of the architecture with the streamlines [212, 219].

## Conclusion and discussion

This review critically examines aerosol filtration in fibrous media by integrating theoretical modeling, numerical simulation, structural reconstruction, experimental validation, and fiber fabrication strategies into a unified analytical framework. While classical single-fiber theories provide essential insight into diffusion, interception, and inertial impaction mechanisms, their assumptions of homogeneity and steady-state flow limit their applicability to realistic, heterogeneous filter structures. In addition, electrostatic interactions, including charge generation, transport, and stability, play a significant role in filtration performance but are often treated independently from mechanical capture mechanisms. Bridging these gaps remains a central challenge in filtration science.

A key limitation identified in the literature is the accurate reconstruction of three-dimensional fibrous geometries for computational analysis. Image-based techniques such as SEM, X-ray tomography, and MRI often fail to capture internal complexity, including void distribution, fiber overlap, and structural heterogeneity, leading to discrepancies between simulated and experimental results. The development of stochastic and hybrid geometry generation methods is therefore essential for improving predictive reliability.

In numerical modeling, current CFD-based approaches frequently rely on simplified assumptions, including laminar and steady-state flow conditions, which neglect transient behavior, turbulence, particle interactions, and dynamic clogging. These limitations restrict the ability of simulations to represent realistic filtration processes. Future work should focus on incorporating unsteady flow, multiscale coupling, and evolving filter structures to enhance predictive accuracy.

Experimental validation remains essential but is constrained by instrumentation limitations and aerosol generation challenges. Measurement uncertainties arising from particle classification methods, coincidence effects, and agglomeration can affect data accuracy. Improved integration of complementary measurement techniques and calibration strategies is necessary to support model validation and reduce discrepancies.

Advances in fiber fabrication techniques, including electrospinning, bubble electrospinning, meltblowing, and supersonic solution blowing, provide significant opportunities for tailoring fiber morphology, porosity, and surface characteristics. However, the relationship between fabrication parameters, resulting microstructure, charge behavior, and filtration performance remains insufficiently quantified. Establishing clear material–process–performance relationships is essential for enabling rational and predictive filter design.

This review emphasizes that filtration performance is governed by the coupled effects of structural parameters, operating conditions, and electrostatic behavior. Factors such as fiber diameter, orientation, humidity, face velocity, and particle concentration interact in complex ways that are not fully captured by current models. In particular, the role of electrostatic forces under varying environmental conditions requires further investigation.

In summary, advancing fibrous aerosol filtration requires an integrated, multiscale approach that connects theory, electrostatic interactions, material properties, and fabrication processes. The unified framework presented in this review provides a pathway toward bridging the gap between idealized models and real-world performance. Future research should focus on developing predictive models that incorporate coupled physical mechanisms and enable the design of high-efficiency, low-resistance, and durable filtration systems.
